# Generation of Sharp Wave-Ripple Events by Disinhibition

**DOI:** 10.1523/JNEUROSCI.2174-19.2020

**Published:** 2020-10-07

**Authors:** Roberta Evangelista, Gaspar Cano, Claire Cooper, Dietmar Schmitz, Nikolaus Maier, Richard Kempter

**Affiliations:** ^1^Department of Biology, Institute for Theoretical Biology, Humboldt-Universität zu Berlin, Berlin, 10115, Germany; ^2^Bernstein Center for Computational Neuroscience Berlin, Berlin, 10115, Germany; ^3^Charité-Universitätsmedizin Berlin (corporate member of Freie Universität Berlin, Humboldt-Universität zu Berlin, and Berlin Institute of Health), Neuroscience Research Center, Berlin, 10117, Germany; ^4^NeuroCure Cluster of Excellence, Charité-Universitätsmedizin Berlin, Berlin, 10117, Germany; ^5^Deutsches Zentrum für Neurodegenerative Erkrankungen in der Helmholtz-Gemeinschaft, Charité-Universitätsmedizin Berlin, Berlin, 10117, Germany; ^6^Einstein Center for Neurosciences Berlin, Berlin, 10117, Germany

**Keywords:** CA3, disinhibition, hippocampus, memory consolidation, sharp wave-ripple complexes

## Abstract

Sharp wave-ripple complexes (SWRs) are hippocampal network phenomena involved in memory consolidation. To date, the mechanisms underlying their occurrence remain obscure. Here, we show how the interactions between pyramidal cells, parvalbumin-positive (PV^+^) basket cells, and an unidentified class of anti-SWR interneurons can contribute to the initiation and termination of SWRs. Using a biophysically constrained model of a network of spiking neurons and a rate-model approximation, we demonstrate that SWRs emerge as a result of the competition between two interneuron populations and the resulting disinhibition of pyramidal cells. Our models explain how the activation of pyramidal cells or PV^+^ cells can trigger SWRs, as shown *in vitro*, and suggests that PV^+^ cell-mediated short-term synaptic depression influences the experimentally reported dynamics of SWR events. Furthermore, we predict that the silencing of anti-SWR interneurons can trigger SWRs. These results broaden our understanding of the microcircuits supporting the generation of memory-related network dynamics.

**SIGNIFICANCE STATEMENT** The hippocampus is a part of the mammalian brain that is crucial for episodic memories. During periods of sleep and inactive waking, the extracellular activity of the hippocampus is dominated by sharp wave-ripple events (SWRs), which have been shown to be important for memory consolidation. The mechanisms regulating the emergence of these events are still unclear. We developed a computational model to study the emergence of SWRs and to explain the roles of different cell types in regulating them. The model accounts for several previously unexplained features of SWRs and thus advances the understanding of memory-related dynamics.

## Introduction

Sharp wave-ripple complexes (SWRs) are brief (50–100 ms) events of elevated and synchronized network activity originating in the CA3 region of the mammalian hippocampus. They occur during periods of awake rest and slow-wave sleep (Buzsáki, 1986, 2015) and have been shown to be critically involved in the process of episodic memory consolidation (Axmacher et al., 2008; Eschenko et al., 2008; Dupret et al., 2010; Girardeau et al., 2014). Sequences of active cells encoding a specific memory are preferentially replayed during SWRs (Wilson and McNaughton, 1994; Skaggs and McNaughton, 1996), and their selective blockage impairs memory performance (Girardeau et al., 2009; Ego-Stengel and Wilson, 2010). The spontaneous emergence of SWRs *in vitro* (Maier et al., 2002, 2003; Hájos et al., 2009) and their persistence after cortical lesions *in vivo* (Buzsáki et al., 1983; Suzuki and Smith, 1988; Bragin et al., 1995) suggest that SWRs are an intrinsic hippocampal phenomenon. Furthermore, *in vitro* SWRs share many properties of *in vivo* SWRs (for review, see Maier and Kempter, 2017), a feature that provides the opportunity to study the hippocampal microcircuit supporting the emergence of SWRs *in vitro*.

Hippocampal cell populations express characteristic activity patterns during SWRs. Pyramidal cells fire sparsely outside SWRs and increase their firing (∼6-fold) during a SWR event (Csicsvari et al., 1999a; Stark et al., 2014). Parvalbumin-positive basket cells (PV^+^ BCs) have been shown to increase their firing activity (∼3-fold) during SWRs (Csicsvari et al., 1999b), while remaining almost silent in non-SWR periods (Csicsvari et al., 1999b; Klausberger and Somogyi, 2008). Bistratified interneurons, oriens-lacunosum-moleculare interneurons, and axo-axonic cells have been shown to increase their firing at different phases of SWR events (Klausberger et al., 2003; Varga et al., 2012, 2014; Hájos et al., 2013; Pangalos et al., 2013; Katona et al., 2014, 2017), whereas other types of interneurons, such as cholecystokinin-positive BCs and ivy cells, seem to be weakly modulated by SWRs (Klausberger et al., 2005; Lasztoczi et al., 2011).

The dynamics of SWR generation is not well understood. It was proposed that SWRs are generated by a buildup of activity in the CA3 area (Buzsáki et al., 1983; de la Prida et al., 2006); this hypothesis was supported by strong recurrent connectivity among CA3 pyramidal neurons (Miles and Wong, 1986; Amaral and Witter, 1989; Ishizuka et al., 1990; Witter, 2007), a result that has been, however, recently challenged (Guzman et al., 2016).

Recent studies have emphasized the involvement of interneurons in the initial phase of SWRs (Ellender et al., 2010; Sasaki et al., 2014; Schlingloff et al., 2014; Bazelot et al., 2016). Schlingloff et al. (2014) specifically showed that a brief (whole-slice) optogenetic activation of PV^+^ cells *in vitro* triggered events identical to spontaneous SWRs, regardless of stimulation length. Additionally, optogenetic silencing of PV^+^ cells interrupted SWR events and strongly decreased the likelihood of observing spontaneous SWRs. How can the early involvement of PV^+^ interneurons be linked to the initiation of a SWR? In this study, we address this question and explain various other features of SWRs using a theoretical approach.

We propose disinhibition as a mechanism that controls the emergence of SWRs in CA3. Disinhibition has been shown to be a ubiquitous feature of cortical circuits (Silberberg and Markram, 2007; Pfeffer et al., 2013; Karnani et al., 2016; Pelkey et al., 2017; for review, see Letzkus et al., 2015). Disinhibitory motifs could also play an important role in the hippocampus (for review, see Chamberland and Topolnik, 2012), for example, in establishing long-lasting memory traces in an object-recognition task (Donato et al., 2013), and in spatial memory tasks in CA1 (Turi et al., 2019). In the context of SWR generation, a disinhibitory mechanism could reconcile, for example, the counterintuitive results of Schlingloff et al. (2014) by hypothesizing that pyramidal cells are disinhibited as a result of PV^+^ cell activation and consequent suppression of another interneuron class.

To evaluate this disinhibition hypothesis, we simulate and analyze minimal computational models of CA3, which reproduce the basic microcircuitry. We first show that SWRs can emerge spontaneously, and that the simulated dynamics mimics the experimental one: SWRs can be elicited by pyramidal or PV^+^ cell stimulation (Schlingloff et al., 2014; Bazelot et al., 2016), and the SWR amplitude is correlated to the intervals between successive SWRs (Kohus et al., 2016; Jiang et al., 2018), which can be explained by short-term depression in the connections emerging from interneurons. Finally, we show that the existence of a bistable configuration in the network is a useful property to better understand the principles governing SWR generation in this type of disinhibitory network. Overall, this study establishes disinhibition as a key network motif in CA3 and sheds light on the possible roles of interneurons in controlling network activity during SWRs.

## Materials and Methods

**Figure 1.**
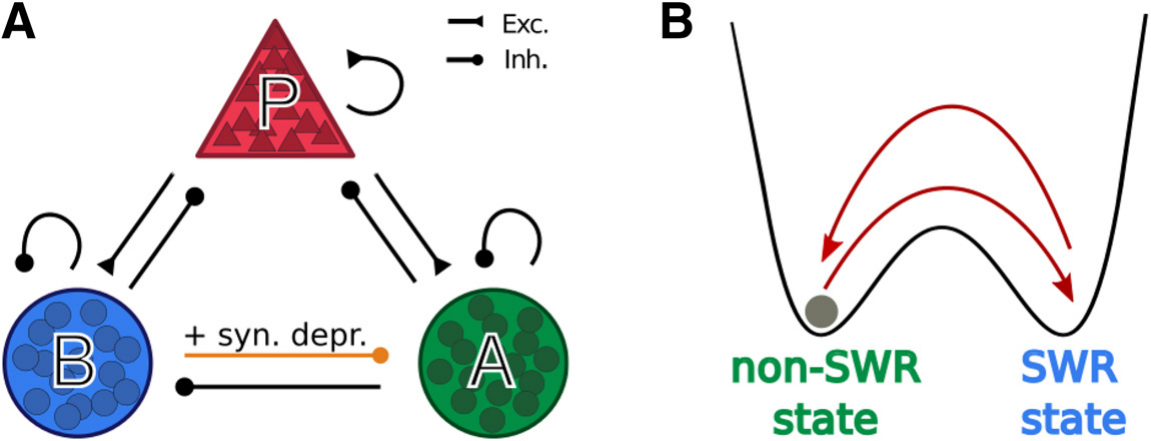
Network structure. ***A***, The network model comprises a population of pyramidal cells (*P*) and two groups of interneurons (PV^+^ BCs and anti-SWR cells, *B* and *A*, respectively). Arrows ending with a triangle indicate excitatory connections (Exc.). Arrows ending with a circle indicate inhibitory connections (Inh.). The connection from PV^+^ BCs to anti-SWR cells includes a short-term synaptic depression mechanism (syn. depr.). ***B***, Schematic representation of network behavior through a particle (gray circle) moving in a potential landscape. The dynamics is characterized by the alternation between non-SWR and SWR states. Text color represents the dominant interneuron type in either state. External factors (current injection or dynamic synaptic depression) can be used to trigger transitions between the two states.

We consider a computational model comprising a population of pyramidal cells (*P* in what follows) and two populations of different types of interneurons: PV^+^ BCs (called *B* in the model) and a class of yet unidentified anti-SWR cells (*A* in what follows). We model neurons as populations of spiking neurons that are recurrently connected as depicted in Figure 1*A*. Furthermore, to be able to perform a mathematical analysis, we also consider a simpler model in which the activity of each of the three populations is described by a firing rate.

As we will show in detail below, in both types of models (spike-based and rate-based), the coexistence of two classes of interneurons in the network (the *B* and *A* cells) allows us to explain, for example, the experimentally observed increase of pyramidal cell firing on activation of *B* cells (Schlingloff et al., 2014): when *B* cells are activated, *A* cells are inhibited, and thus release the inhibition of *P* cells. This interaction can result in an increase in the firing of *P* and *B* cells and a decrease in the firing of *A* cells. We interpret this pattern of activation, in which *P*, *B*, and *A* simultaneously change their firing rates (from low activity to high activity for *P* and *B* cells, and from high activity to low activity for *A* cells), as a signature of the initiation of a SWR event. A SWR terminates when the high activity of *A* cells is restored, and the activity of *P* and *B* cells is low; this firing pattern is characteristic of the non-SWR state.

In what follows in Materials and Methods, we first describe networks of spiking neurons and then define and analyze rate models.

### Spiking model

#### Neuron model

To keep models of spiking networks as simple as possible, neurons are described as conductance-based leaky integrate-and-fire units. The subthreshold membrane potential $V_{i}(t)$ of cell *i* obeys

(1) $$C\frac{\text{d}V_{i}}{\text{d}t}=g_{L}(V_{rest}-V_{i})-[g_{i}^{P}(t)(V_{i}-E_{rev}^{P})+g_{i}^{A}(t)(V_{i}-E_{rev}^{A})+g_{i}^{B}(t)(V_{i}-E_{rev}^{B})]+I_{ext}$$

where *C* = 200 pF is the membrane capacitance and *g_L_* = 10 nS is the leak conductance, resulting in a membrane time constant τ = 20 ms. *V_rest_* = −60 mV is the resting membrane potential, $E_{rev}^{P}=0$ mV, $E_{rev}^{B}=-70$ mV, and $E_{rev}^{A}=-70$ mV are the reversal potentials of excitation and inhibition (of *B* and *A* cells, respectively), and $I_{ext}=I_{BG}+I_{i}$ is the sum of external currents. To elicit activity in the network, a constant background current *I_BG_* = 200 pA is injected to all neurons. Only if explicitly mentioned, neurons receive additional time-dependent currents *I_i_*. Every time a neuron's membrane potential reaches the threshold *V_thr_* = –50 mV, a spike is emitted and *V_i_* is reset to the reset potential (for simplicity, it equals *V_rest_*), where it is clamped for a refractory period of length $t_{refr}^{I}=1$ ms, I∈{P,B,A}. These and further neuronal parameters are summarized in Table 1.

**Table 1.** Intrinsic neuronal parameters for the spiking network (used in Figs. 2-4, 9, 11, 13-15)

Parameter	Value	Definition
*N_P_*	8200	No. of pyramidal cells (*P*)
*N_B_*	135	No. of PV^+^ BCs (*B*)
*N_A_*	50	No. of anti-SWR cells (*A*)
$\tau_{syn}^{P}$	2 ms	Glutamatergic synaptic time constant
$\tau_{syn}^{A}$	4 ms	GABAergic synaptic time constant (*A* cells)
$\tau_{syn}^{B}$	1.5 ms	GABAergic synaptic time constant (*B* cells)
*g_L_*	10 nS	Leak conductance
*V_rest_*	−60 mV	Resting potential
*V_thr_*	−50 mV	Voltage threshold
$E_{rev}^{P}$	0 mV	Excitatory reversal potential
$E_{rev}^{A}$	−70 mV	Inhibitory (*A*) reversal potential
$E_{rev}^{B}$	−70 mV	Inhibitory (*B*) reversal potential
*C*	200 pF	Membrane capacitance
$t_{refr}^{P}$	1 ms	Refractory period *P*
$t_{refr}^{B}$	1 ms	Refractory period *B*
$t_{refr}^{A}$	1 ms	Refractory period *A*
*I_BG_*	200 pA	Constant background current

The outgoing synapses from pyramidal cells are modeled as fast AMPA-type synapses, and the synapses originating from *B* or *A* cells are modeled as GABA_A_-type synapses (for motivation, see, e.g., Ellender et al., 2010). The time-dependent variables $g_{i}^{P}(t),\;g_{i}^{B}(t)$, and $g_{i}^{A}(t)$ describe the total synaptic conductances resulting from incoming synaptic inputs to neuron *i*. To simplify the notation, the explicit time dependence is dropped. The conductance dynamics are described by the following:

(2) $$\begin{array}{ll} & \frac{\text{d}g_{i}^{P}}{\text{d}t}=-\frac{g_{i}^{P}}{\tau_{syn}^{P}}+\sum_{f,j}\delta (t-t_{j}^{\left(f\right)}-\tau_{IP})g_{ij}^{IP}\\ & \frac{\text{d}g_{i}^{B}}{\text{d}t}=-\frac{g_{i}^{B}}{\tau_{syn}^{B}}+\sum_{f,j}\delta (t-t_{j}^{\left(f\right)}-\tau_{IB})g_{ij}^{IB}\\ & \frac{\text{d}g_{i}^{A}}{\text{d}t}=-\frac{g_{i}^{A}}{\tau_{syn}^{A}}+\sum_{f,j}\delta (t-t_{j}^{\left(f\right)}-\tau_{IA})g_{ij}^{IA},\;I\in \{P,B,A\} \end{array}$$

where $\delta (t-t_{j}^{\left(f\right)}-\tau_{IJ})$ is the contribution of the *f*-th incoming spike (from neuron *j* at time $t_{j}^{\left(f\right)}$); δ is the Dirac δ function. The quantities $g_{ij}^{IP},\;g_{ij}^{IB}$, and $g_{ij}^{IA}$ describe the unitary conductance increases resulting from a single spike. For example, $g_{ij}^{IP}$ is the conductance increase by presynaptic neuron *j* in population *P* connected to postsynaptic neuron *i* in population I∈{P,B,A} (i.e., these values depend on the synapse type). There is a delay between a presynaptic spike and the postsynaptic response onset defined as $\tau_{IJ}=1$ ms for all synapse types. The conductances decay exponentially with time constants $\tau_{syn}^{P}=2$ ms, $\tau_{syn}^{B}=1.5$ ms, and $\tau_{syn}^{A}=4$ ms (Geiger et al., 1995; Bartos et al., 2002; Taxidis et al., 2012). For simplicity, time constants only depend on the presynaptic but not the postsynaptic type. The values of the unitary conductance increases are assumed to be the same for all synapse pairs *i*, *j* from population *J* to population *I*. They range from 0.05 to 8 nS; these values and further synaptic parameters are listed in Table 2.

**Table 2.** Synaptic and connectivity parameters for the spiking network (used in Figs. 2–4, 9, 11, 13–15)*^a^*

Connection	Connection probability	Conductance increase (nS)	Synaptic delay (ms)
*P* → *P*	$p^{PP}=0.01$	$g^{PP}=0.2$	$\tau_{PP}=1$
*P* → *A*	$p^{AP}=0.01$	$g^{AP}=0.2$	$\tau_{AP}=1$
*A* → *A*	$p^{AA}=0.6$	$g^{AA}=4$	$\tau_{AA}=1$
*A* → *P*	$p^{PA}=0.6$	$g^{PA}=6$	$\tau_{PA}=1$
*P* → *B*	$p^{BP}=0.2$	$g^{BP}=0.05$	$\tau_{BP}=1$
*B* → *B*	$p^{BB}=0.2$	$g^{BB}=5$	$\tau_{BB}=1$
*B* → *P*	$p^{PB}=0.5$	$g^{PB}=0.7$	$\tau_{PB}=1$
*A* → *B*	$p^{BA}=0.6$	$g^{BA}=7$	$\tau_{BA}=1$
*B* → *A*	$p^{AB}=0.2$	$g^{AB}=8$	$\tau_{AB}=1$

*^a^*More details are provided in Spiking model. *g^AB^* does not include the contribution of short-term synaptic depression.

#### Numbers of cells

We model a network comprising *N_P_* = 8200 pyramidal cells (*P*), *N_B_* = 135 PV^+^ BCs (*B* in the model), and *N_A_* = 50 anti-SWR cells (*A*) cells. These numbers are chosen to mimic the number of *P* and *B* cells present in CA3 in a 400-μm-thick rat slice. It has been estimated that the entire rat hippocampus contains 204,700 pyramidal cells and 25,300 interneurons in the CA3 region (Bezaire and Soltesz, 2013). Given that a 400-μm-thick slice represents ∼4% of the volume of the rat hippocampus, we estimate that ∼8200 pyramidal cells are present in a slice. In CA1, PV^+^ BCs are thought to account for ∼14% of all interneurons. As we do not have a closer estimate for CA3, we assume the same holds in CA3, yielding ∼135 PV^+^ BCs in a CA3 slice. Given that the identity of anti-SWR cells is unknown, no such data are available for these cells; we decided to include 50 anti-SWR cells in the network. In our model, the *P*, *B*, and *A* cells are assumed to be homogeneous groups, which tremendously facilitates the model setup and makes an analysis practicable. Thus, here we do not distinguish between cells that are participating in a SWR and those that are not.

#### Connectivities

Neurons are randomly connected with connection probability *p^IJ^* for connection J→I. In contrast to the dominant view of CA3 as a strongly recurrent region, it was recently shown that CA3 pyramidal cells are, at least *in vitro*, only sparsely connected (Guzman et al., 2016). We thus choose *p^PP^* = 0.01. Recurrent connectivity among PV^+^ BCs is usually estimated to be ∼20% in rat CA1 (Sik et al., 1995; Donoso, 2016) and in mouse CA3 (Schlingloff et al., 2014); a recent study (Kohus et al., 2016) suggested that connectivity could be as high as 66% (in mouse CA3, *in vitro*); nevertheless, we consider the conservative estimate of 20% and thus set *p^BB^* = 0.2. A large body of work studies the bidirectional connectivity between pyramidal cells and interneurons; however, only few studies are specific for PV^+^ cells (possibly BCs); and, to our knowledge, none of these addresses CA3. Mouse CA1 studies (Lee et al., 2014) suggest that the connectivity from PV^+^ BCs to pyramidal cells could be in the range of 45%-50%, and the one from pyramidal cells to PV^+^ BCs is ∼16%-48%. We choose *p^PB^* = 0.5 and *p^BP^* = 0.2.

For the connectivity from and to anti-SWR cells, we choose the values: $p^{AP}=0.01,\;p^{PA}=0.6,\;p^{AA}=0.6,\;p^{AB}=0.2$, and *p^BA^* = 0.6. These values are in line with experiments showing that the connectivities between principal cells and interneurons, as well as connectivities among interneurons, are distributed in the range 0%-90%: for hippocampus (Böhm et al., 2015; Kohus et al., 2016; Pelkey et al., 2017; Booker and Vida, 2018); for neocortex (Kwan and Dan, 2012; Walker et al., 2016; Riedemann, 2019). Future information about the identity of anti-SWR cells will help refining the connectivity values. The value chosen for connectivities from the population of 50 *A* cells to the other populations ($p^{PA}=p^{BA}=p^{AA}=0.6$) imply that each neuron in the postsynaptic population receives, on average, 50 × 0.6 = 30 synapses from the presynaptic *A* population. In general, it has been shown that as long as this number of synapses is much larger than 5, the behavior of a network does not critically depend on connectivity, but more on the product of connectivity and efficacy of the synapses (Chenkov et al., 2017). These results and our numerical analysis of the network dynamics of rate models indicate that a large number of parameter combinations reproduces the desired network behavior, suggesting that the exact values of the connectivities do not impact the main model outcomes. All connectivity parameters are listed in Table 2.

#### Short-term plasticity

A short-term synaptic depression mechanism is assumed to be present at the B→A connections, which modulates the strength of the unitary synaptic conductance increases. The synaptic increases $g_{ij}^{AB}$ from neuron *j* in population *B* to neuron *i* in population *A* are scaled by a factor $e_{ij}^{AB}$ describing the synaptic efficacy, which evolves over time as follows:

(3) $$\frac{\text{d}e_{ij}^{AB}}{\text{d}t}=\frac{1-e_{ij}^{AB}}{\tau_{D}}-\sum_{f}\delta (t-t_{j}^{\left(f\right)})e_{ij}^{AB}\eta_{D}.$$

Every time a cell *j* in population *B* spikes, the $g_{i}^{B}$ conductance for the connected postsynaptic cells *i* is increased by the product $e_{ij}^{AB}g_{ij}^{AB}$ (instead of only $g_{ij}^{AB}$ as in the nondepressed case, see Eq. 2), and the $e_{ij}^{AB}$ variables of all synapses starting from the spiking cell *j* are decreased by an amount $e_{ij}^{AB}\eta_{D}$. Hence, higher activity (i.e., more spikes per second) of one cell in population *B* results, on average, in a lower efficacy of synaptic transmission to its connected cells in population *A*. To prevent the emergence of negative conductance changes, $e_{ij}^{AB}$ is restricted to the interval [0, 1] through the dynamics described in Equation 3. The depression mechanism, with values chosen as η*_D_* = 0.18 and τ*_D_* = 250 ms (see also Table 3), is responsible for the termination of a SWR event and, more in general, for driving the system back to the non-SWR state. In Results, the synaptic efficacy variable *e^AB^* defines the averaged value of $e_{ij}^{AB}$ across all B→A synapses.

**Table 3.** Synaptic depression and facilitation parameters used to simulate the spiking model (Figs. 2–4, 9, 11, 13, 14)*^a^*

Parameter	Value	Definition
η_D_	0.18	Depression rate of connection B→A
τ_D_	0.25 s	Synaptic depression time constant of connection B→A
η_F_	0.15	Facilitation rate of connection P→A
τ_F_	0.25 s	Synaptic facilitation time constant of connection P→A
z_max_	1	Upper bound for increase in facilitation of connection P→A

*^a^*For the simulations in Figure 15, we use $\eta_{F}=0.32$ and $\tau_{F}=230$ ms. For details, see Eqs. 3, 4.

If not explicitly mentioned, all other conductance increases $g_{ij}^{IJ}$ are kept fixed. In specific cases (see Additional short-term plasticity mechanisms), the P→A connection is considered to be plastic, with a short-term facilitation mechanism, and the B→P has a short-term depression mechanism analogous to the one described above. For the latter case, the $g_{ij}^{PB}$ conductance is scaled by a synaptic efficacy variable analogous to what is described by Equation 3. For the simulations with additional B→P synaptic depression, we choose η*_D_* = 0.18 and τ*_D_* = 250 ms, analogous to the B→A depression. All other parameters are unchanged.

The facilitation at the synapses P→A in Additional short-term plasticity mechanisms is modeled as follows: the variable $z_{ij}\geq 0$ describes the synapse-specific effect of facilitation. In the case of no facilitation, *z_ij_* = 0. The facilitation variables evolve over time as follows:

(4) $$\frac{\text{d}z_{ij}}{\text{d}t}=-\frac{z_{ij}}{\tau_{F}}+\sum_{f}\delta (t-t_{j}^{\left(f\right)})(z_{max}-z_{ij})\eta_{F}.$$

**Figure 2.**
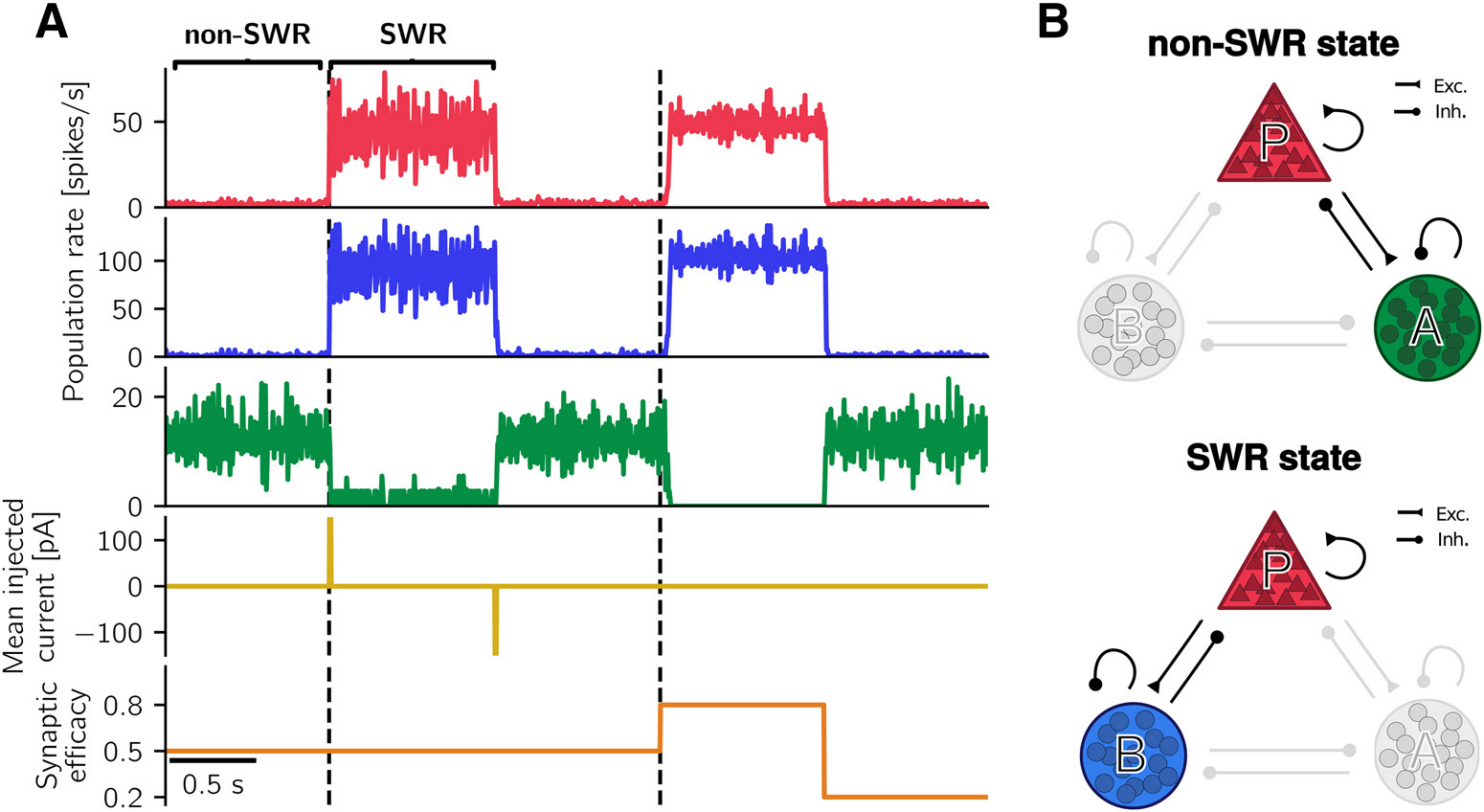
The spiking network is bistable for intermediate, fixed synaptic efficacy. ***A***, Simulation results as synaptic efficacy is clamped at different values. For *e^AB^* = 0.5 (average value of synaptic efficacies of synapses B→A), two stable states exist. Depolarizing-current injection to *P* cells can switch the system from the non-SWR state to the SWR state. Hyperpolarizing current to *P* cells restores the non-SWR state. Population rates for the non-SWR state are as follows: *P* = 1.94 spikes/s, *B* = 1.32 spikes/s, *A* = 12.56 spikes/s; and during the SWR state as follows: *P* = 43.60 spikes/s, *B* = 91.87 spikes/s, *A* = 1.12 spikes/s. After a switch of *e^AB^* from 0.5 to 0.8, the network jumps to the SWR state because of internal fluctuations. There is a small delay with respect to simulation start (right black dashed line), compared with the nearly instantaneous jump in the case of current injection and *e^AB^* = 0.5 (left black dashed line). The non-SWR state is restored for *e^AB^* = 0.2. Parameters used to simulate the spiking network are listed in Tables 1–3. ***B***, Schematic of the dominant subnetworks in non-SWR and SWR states (annotations as in Fig. 1*A*). Top, non-SWR state: the interaction between *P* and *A* cells governs the network, whereas *B* cells are almost inactive. Despite the low firing rate of *P* cells, their inputs to *A* cells are needed to keep *A* cells active. Bottom, SWR state: active *P* and *B* cells dominate the network, whereas *A* cells are almost inactive.

Every time a cell *j* in the *P* population spikes, the AMPA conductance $g_{ij}^{AP}$ of a connected cell *i* (see Eq. 2) is scaled by a factor $\left(1+z_{ij}\right)$, and the *z_ij_* variables of all synapses *i* whose presynaptic cell is *j* are increased by an amount $(z_{max}-z_{ij})\eta_{F}$. The value *z_max_* is a constant defining an upper bound for the increase in facilitation. When the system is in the non-SWR state, *z_ij_* decays exponentially to the average value $z_{\text{non-SWR}}=\frac{P_{0}z_{max}\eta_{F}\tau_{F}}{1+P_{0}\eta_{F}\tau_{F}}$, where *P*_0_ is the firing rate of *P* cells in the non-SWR state (∼2 spikes/s; see Fig. 2*A*). To be able to better compare the default network (with only B→A depression) to the case where extra facilitation is added, we additionally normalize $g_{ij}^{AP}$ by dividing it by $\left(1+z_{\text{non-SWR}}\right)$. This assures that when the facilitation is active, but has reached $z_{\text{non-SWR}}$, the P→A synapses have the same average strength (i.e., the same conductances) as in the model with no facilitation. For the simulations with additional P→A synaptic facilitation, we choose $\eta_{F}=0.15,\;\tau_{F}=250$ ms, *z_max_* = 1. All the other parameters are as in the default model. For the simulation where P→A facilitation is the only plastic mechanism, we need to adjust the parameters for the network to be in a regimen where the non-SWR state is destabilized and events can start spontaneously with a large enough incidence (if the incidence is too low, we cannot observe correlation between interevent interval [IEI] and event amplitude). To this end, we choose *g^AB^* = 4.5 nS, *g^BA^* = 5.5 nS, τ*_F_* = 230 ms, η*_F_* = 0.32, *z_max_* = 1, and do not normalize *g^AP^* by its non-SWR state value ($z_{\text{non-SWR}}$). *g^AB^* and *g^BA^* have to be decreased in the P→A facilitation-only scenario: with default values and fixed B→A synaptic efficacy at $e^{AB}=0.5$, the system would stay in the SWR state because the facilitation effect would be counterbalanced by a too strong B→A connection.

#### Desired firing rates

To construct the spiking network (Fig. 1*A*), whose dynamics is shown in Figure 2 and in Results, we aim to set the connections among the different populations such that the simulated firing rates of *P*, *B*, and *A* cells match the desired firing rates of pyramidal cells, PV^+^ BCs, and anti-SWR cells, respectively. Briefly, experimentally observed firing rates for pyramidal cells in non-SWR periods are in the range of 0.03-3 spikes/s, and in the range of 1-13 spikes/s for SWR periods (Ylinen et al., 1995; Klausberger et al., 2003; Lapray et al., 2012; Hájos et al., 2013; English et al., 2014), although they can reach 40 spikes/s (English et al., 2014). Firing rates of PV^+^ BCs are in the range of 2–20 spikes/s in non-SWR periods and up to ∼120 spikes/s during SWRs (Klausberger et al., 2003; Lapray et al., 2012; Varga et al., 2012; Hájos et al., 2013). We assume that anti-SWR cells fire ∼12 spikes/s in non-SWR states and are almost silent during SWRs (firing rate ∼1 spike/s).

The network is constructed such that, in the non-SWR state, the *P* and *A* populations are in an asynchronous irregular (AI) regimen, which could reflect the state of CA3 at rest (Ikegaya et al., 2013). In this state, population firing rates are tuned to have *P* cells firing at ∼2 spikes/s (i.e., ∼16,400 spikes/s in total for the whole population), *A* cells at ∼12 spikes/s (i.e., ∼600 spikes/s in total), and *B* cells to be almost inactive, with average firing rates at ∼1 spike/s (i.e., ∼135 spikes/s in total). The SWR state is dominated by a strongly active *P-B* subnetwork, where *P* cells fire at 43 spikes/s, *B* cells fire at ∼90 spikes/s, and *A* cells are almost inactive, with average firing rates at ∼1 spike/s. Because we have assumed that *P* cells are a homogeneous population, the chosen average firing rate of 43 spikes/s in the SWR state is larger than what is observed as an average value in experiments. However, as motivated further below in this section, the particular value of the firing rate is not important as long as it is well above the spontaneous rate. We nevertheless use the value of 43 spikes/s here to accentuate the highly active SWR state.

#### Requirements on pathway strengths

As also discussed in Rate model and in Results, the relative strengths of the incoming pathways to a given population need to be adjusted to guarantee that cell stimulation yields SWR events that are similar to experimentally recorded SWRs.

Crucially, the disynaptic pathway B→A→P should be stronger than the direct connection B→P for the activation of *B* cells to result in an increase of pyramidal cells firing. In summary, requirements on converging pathways in the network of Figure 1 are as follows:

1. Pathway P→B→A should be stronger than P→A. This guarantees that the activation of *P* decreases *A* firing.
2. Pathway P→B should be stronger than P→A→B. This guarantees that the activation of *P* increases *B* firing.
3. Pathway B→A→P should be stronger than B→P. This guarantees that the activation of *B* increases *P* firing (i.e., it activates the disinhibition mechanism).
4. Pathway A→P should be stronger than A→B→P. This guarantees that the inactivation of *A* increases *P* firing.

The enforcement of the requirements 1-4 guarantees that, on cell stimulation, the firing rates of all populations change as desired. Two additional sets of converging pathways exist in the network: (1) the pathways B→A and B→P→A; and (2) the pathways A→B and A→P→B. However, pathways in (1) collaborate to decrease the activation of *A*, and pathways in (2) collaborate to increase the activation of *B* on inactivation of *A*; thus, no requirements need to be enforced. Indeed, these conditions demand that at least one of two pathways (B→A and B→P→A, and A→B and A→P→B, respectively) is strong enough for a current injection to elicit the desired response, but these requisites are already included in the requirements 1-4 (e.g., a sufficiently strong B→A is included in requirements 1 and 3).

The strength of a pathway is a combination of the average connection strength (which in turn depends on the connection probability, the size of the presynaptic population, and the contribution of a single incoming postsynaptic potential) and the input-output relation of the postsynaptic population (for a more formal way of defining these pathway strengths, see Bifurcation analysis of rate model). In formulating these requirements, we are implicitly incorporating the recurrences of the populations (e.g., the recurrent *A* connection in the pathway B→A→P), and we are neglecting any temporal structure (delays) in the network.

#### Constructing the spiking network

To construct a network, we start by fixing the numbers of cells and the connection probabilities of *P*, *B*, and *A* cells using the values already introduced (Tables 1 and 2). To tune the values of the unitary conductance increases $g_{ij}^{IJ}$, for I,J∈{P,B,A}, we rely on the observation that the two groups of interneurons *B* and *A* should be active at different stages. *B* cells should be almost inactive in non-SWR states, and have high firing rates during SWRs, whereas *A* cells should be tonically active throughout the non-SWR state and stop firing during the SWR-state. Thus, both the non-SWR and SWR states are dominated by a subnetwork of active cells: the pyramidal cells, and only one type of interneuron. On a first approximation, we consider the firing rate of the other, nondominant interneuron type as being close to 0 spikes/s.

For this reason, we first construct the network starting from the *P-A* subnetwork in isolation. We assume that the unitary conductance increases $g_{ij}^{IJ}$ are the same across each *i*, *j* combination (i.e., they only depend on the synapse type), and choose the values $g_{ij}^{PP},\;g_{ij}^{AP},\;g_{ij}^{PA}$, and $g_{ij}^{AA}$ such that the neurons in both populations fire asynchronously and irregularly (AI regimen), with mean firing rates P≈2 spikes/s and A≈12 spikes/s. These firing rates have been chosen to be close to experimental values, but the exact choice of the target values does not impact the results presented here. We choose conductance increases values $g_{ij}^{AP}=0.2$ nS, $g_{ij}^{PA}=6$ nS, $g_{ij}^{AA}=4$ nS, and $g_{ij}^{PP}=0.2$ nS. While choosing these values, we enforce the conditions on the pathway strengths by selecting a small enough $g_{ij}^{AP}$ (requirements 1 and 2) and a large enough $g_{ij}^{PA}$ (requirements 3 and 4). These requests are relatively easy to fulfill because $g_{ij}^{PA}$ is expected to be large for the inhibition to stabilize the *P*-*A* subnetwork; and, vice versa, *A* cells should not receive too much excitation. The chosen values of the conductance increases also give rise to irregular and asynchronous firing (AI state), as can be seen by monitoring the coefficient of variation (CV) and the SD of the instantaneous population rates (Gaussian filter time constant is 3 ms) (Vogels et al., 2011): in the *P*-*A* network, neurons fire fairly irregularly (CV > 0.5) and asynchronously (SD < 1 spike/s).

Similarly to the *P*-*A* subnetwork, we then focus on the isolated *P*-*B* subnetwork and tune the conductance-increase values $g_{ij}^{PB},\;g_{ij}^{BP}$, and $g_{ij}^{BB}$ such that *P* cells fire close to P≈43 spikes/s, and *B* cells close to *B* ≈ 90 spikes/s. We use the value of $g_{ij}^{PP}$ defined in the subnetwork *P*-*A*. Also in this case, firing rates have been chosen to be close to experiments, but other choices are also possible. Nevertheless, the firing rates of *P*, *B*, and *A* cells should be sufficiently different in the SWR and non-SWR states (at least ∼ 5 spikes/s difference) for the system to jump between clearly distinguishable states. As a result, conductance increase values are $g_{ij}^{BP}=0.05$ nS, $g_{ij}^{PB}=0.7$ nS, and $g_{ij}^{BB}=5$ nS. Requirements on the strengths of pathways are enforced by selecting a sufficiently large $g_{ij}^{BP}$ (requirements 1 and 2) and a small $g_{ij}^{PB}$ (requirements 3 and 4). Actually, the choice of $g_{ij}^{PB}$ is a compromise between these requirements and the fact that the connection B→P should be strong enough for the interneurons to control the spiking of *P* cells. As a result, it is difficult to obtain a network in an AI state: the units are firing regularly (CV < 0.1) and in synchrony (SD > 1 spike/s). The synchronicity of unit firing is clearly visible in the power spectrum (the peak oscillation frequency is 135 Hz) and results in the ripple-like oscillations in the simulations described in SWRs can be generated in a CA3-like spiking network. The strength and the delay of the recurrency among interneurons as well as the feedback loop between interneurons and excitatory cells regulate the frequency of oscillation (Brunel and Wang, 2003; Donoso et al., 2018).

Up to this point, we have built two subnetworks that display clearly distinguishable states of stable firing of *P* and *A*, and *P* and *B* cells, respectively. We now wish to connect the two subnetworks by defining the reciprocal connections between the interneurons. First, we add *A* cells to the highly active *P*-*B* subnetwork, with the connections A→A and P→A from the *P*-*A* subnetwork simulations, and define a new connection B→A with $g_{ij}^{AB}=8$ nS, such that *A* cells fire at ∼1 spike/s (i.e., are almost inactive in this state). The “disinhibitory” connection B→A is expected to be strong to control the firing of *A* cells and to comply with requirements 1 and 3. This scenario is constructed to represent the SWR state, where we assume that the neglected connection A→P and the not yet defined connection A→B play a negligible role because *A* cells are almost inactive.

As a next step, we simulate a network with all the connections defined in the previous steps, add the new connection A→B, and choose $g_{ij}^{BA}=7$ nS, such that *B* cells fire close to 1 spike/s when the *P*-*A* subnetwork is highly active. This value of the conductance increase is a compromise between the requirements 2 and 4 (which suggest that the connection A→B should be weak enough) and the fact the connection A→B should be strong enough for *B* cells to be inhibited. This scenario corresponds to the non-SWR state.

The full network constructed with this procedure has two embedded stable states: one dominated by the *P*-*A* subnetwork (non-SWR state) and one dominated by the *P*-*B* subnetwork (SWR state). Thus, there is an intrinsic bistability structure in the network: external mechanisms (e.g., current injection) can be used to switch between the two states. The conductance increase values $g_{ij}^{AB}$ and $g_{ij}^{BA}$ regulate the stability of the two states. They are chosen to be large enough to inhibit the inactive interneuron type in each state, but should not be too large, so as to guarantee that both states are stable. For example, even when initialized to be in the non-SWR state, a network with a too strong B→A connection would spontaneously jump to the SWR state. This is because the low activity of the *B* cells is amplified by a strong enough B→A connection that suffices to inhibit the *A* cells.

To generate a network exhibiting spontaneous SWRs, we destabilize the two stable states by modifying the conductance-increase value $g_{ij}^{AB}$: increasing the strength of the B→A connection promotes the inhibition of *A* cells by *B* cells and thus favors the initiation of spontaneous events. Moreover, to allow for spontaneous jumps from the SWR to the non-SWR state, we add a synaptic depression mechanism at the B→A synapses (with dynamics described by Eq. 3), which is responsible for the termination of the SWR state.

Together with the choice of the reciprocal connections among interneurons, the depression parameters τ*_D_* and η*_D_* allow fluctuations in the activity of *B* cells to start a SWR event. In particular, τ*_D_* should be larger than the duration of a SWR event (τ_D_ ≫ 100 ms), but smaller than the average IEI between SWRs (τ_D_ ≪ 1000 ms), and η*_D_* should be such that multiple spikes of the *B* cells are needed to terminate a SWR. As *B* cells fire at ∼90 spikes/s, we expect a *B* cell to fire, on average, 5 spikes/SWR. Furthermore, the existence of spontaneous SWRs with a correlation structure as shown in Features of spontaneous and evoked SWRs match experimental results is controlled by the interplay of the parameters $g_{ij}^{AB},g_{ij}^{BA}$, τ*_D_*, and η*_D_*. We took these aspects into account to choose the values of τ*_D_* and η*_D_* (τ*_D_* = 250 ms, η*_D_* = 0.18). In summary, the synaptic parameters used to simulate the default spiking network are listed in Tables 2 and 3.

#### Simulation analysis

All simulations are performed in Brian (Goodman and Brette, 2009), and data analysis is performed in Python (www.python.org). Population firing rates are computed by averaging the instantaneous firing rates, averaged across neurons, with a Gaussian smoothing window with width 3 ms.

We use the modulation of population firing rates as a signature of a SWR event: an increase of *P* cells firing to ≈ 43 spikes/s, an increase of *B* cells firing to ≈ 90 spikes/s, and a decrease of *A* cells to values <2 spikes/s mark the start of a SWR event. All conditions have to be simultaneously fulfilled for a SWR event to be detected.

To trigger a SWR event, we randomly select 60% of the cells in a given population and stimulate them with currents uniformly distributed between *I* = 0 pA and a maximal value *I_P_* = 300 pA, *I_B_* = 500 pA, or *I_A_* = –500 pA for intervals of length 10 ms. The short stimulation times are comparable with the duration of optogenetic stimulation used in experiments (Schlingloff et al., 2014; Kohus et al., 2016). Stimulation results are hardly affected by differences in the stimulation parameters, as long as the stimulation paradigm is sufficient to initiate a SWR event.

In all simulations shown in this article, the dynamic variables of Equations 1 and 2 (*V_i_*, $g_{i}^{P},\;g_{i}^{B},\;g_{i}^{A}$) are initialized for the system to be in the non-SWR state.

#### Defining the local field potential (LFP) signal

To define the LFP in stratum pyramidale, we assume that the main contribution to the field is provided by perisomatically targeting interneurons (Beyeler et al., 2013; Schönberger et al., 2014), namely, PV^+^ BCs, targeting the cell bodies of pyramidal cells. A main criticism to this approach is that the cell morphology and nonperisomatic (e.g., dendritic) inputs might also contribute (Einevoll et al., 2013; Chizhov et al., 2015). However, a detailed description of the LFP (see e.g., Schomburg et al., 2012; Ramirez-Villegas et al., 2018) is beyond the scope of the simplified point neuron scenario considered here because of its computational complexity (only 150 CA3 cells were simulated in Ramirez-Villegas et al., 2018). Therefore, we resort to this simple approach to define an approximated LFP trace. We believe that multicompartmental models, which would critically rely on the unknown dendritic locations of synapses from *A* cells, would not improve the description of the LFP.

**Figure 3.**
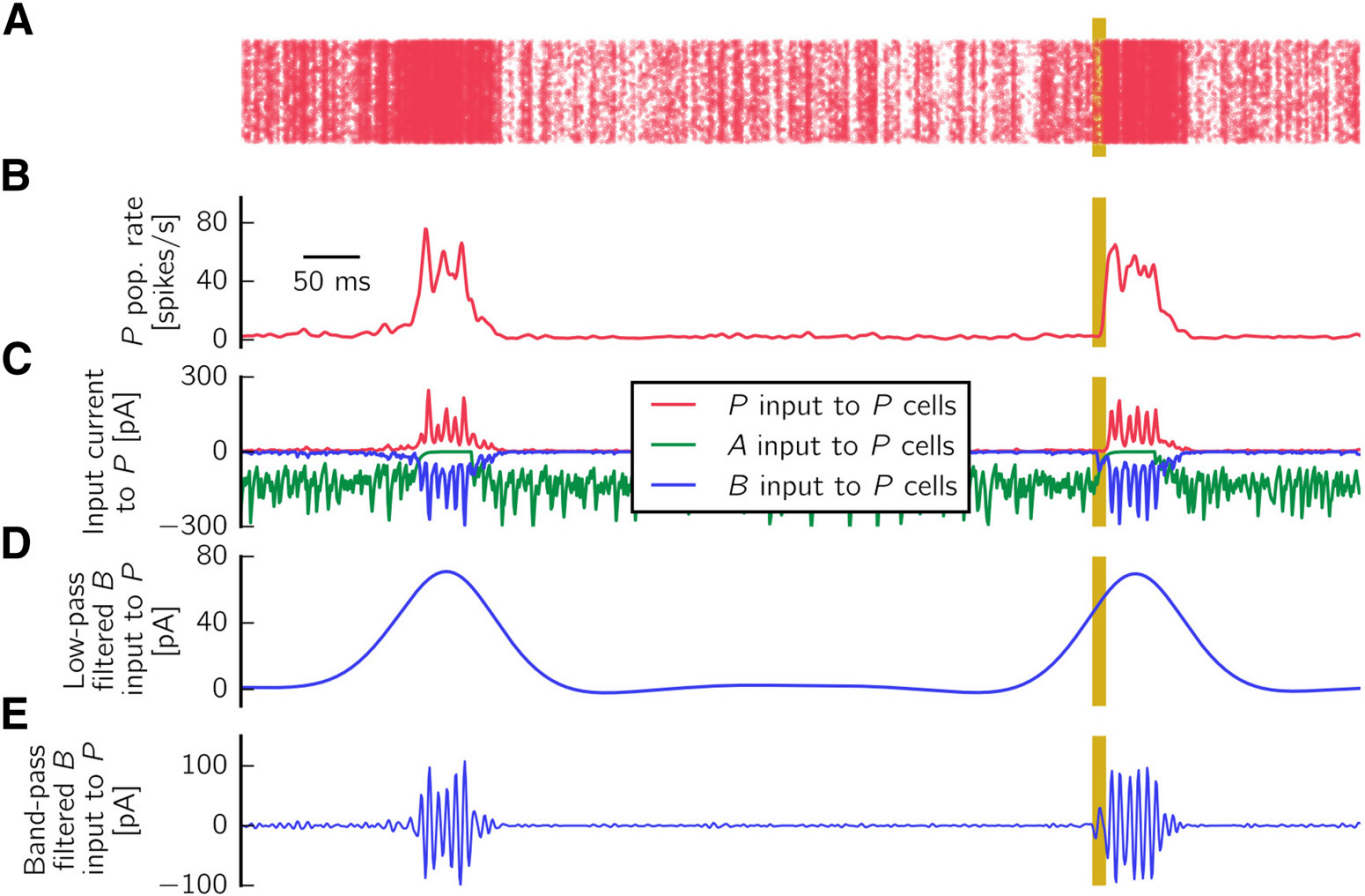
Definition of approximated LFP. ***A***, Raster plot showing the population rate (pop. rate) of *P* cells during a spontaneous and an evoked SWR event. Yellow bars represent the interval of length 10 ms during which current is injected to *B* cells. ***B***, Average population firing rate of *P* cells. ***C***, Input current from *P* (red), *B* (blue), and *A* (green) cells impinging onto pyramidal cells, averaged across all neurons. The averaged input current from *B* to *P* cells is sign-reversed and used as an approximation of the LFP. ***D***, LFP signal shown is low-pass filtered up to 5 Hz to extract the sharp wave component. ***E***, LFP is bandpass filtered in the 90-180 Hz range to extract the ripple component.

We implicitly assume that anti-SWR cells do not contribute to the LFP. This assumption could hold if anti-SWR cells target pyramidal cells at the distal dendrites, so that their contribution at the pyramidal cells somata can possibly be neglected. Notably, Figure 3 shows that there are almost no anti-SWR-cell-related currents impinging onto pyramidal cells during SWRs (our events of interest) because most *A* cells are inactive. Thus, we entirely focus on the contribution from PV^+^ BCs to define the LFP.

In summary, we define the LFP as a filtered version of the synaptic input current from *B* to *P* cells, sign-reversed and averaged over all B→P synapses. To obtain the sharp wave and ripple components of a SWR event, we filter the sign-reversed mean *B* input current to *P* cells in two different frequency bands, using a Butterworth filter of order 2. The sharp wave component is obtained by low-pass filtering the signal up to 5 Hz. The cutoff frequency is chosen for the filter to cover the whole duration of a postsynaptic event. The ripple component is obtained by bandpass filtering the signal in the range 90-180 Hz, around the peak frequency of 135 Hz (the peak is computed in the power spectrum).

#### Quantification of SWR properties

For spontaneous and evoked SWR events, we define the following properties: IEI, amplitude, and the full width at half maximum (FWHM). The amplitude is the peak value of the sharp wave (SW) component, that is, the low-pass filtered LFP signal; peaks are detected using a script available at Duarte (2015). To compute the FWHM, we first define the mean baseline value as the mean across all events of the average value of the low-pass filtered signal in periods preceding a sharp wave by 200-100 ms. Then, we calculate the half maximum by finding the mean value of the event amplitude and the mean baseline value, for each event. We define the start of a sharp wave event as the time of the sharp wave signal at half maximum preceding the peak, and the event end as the time of the sharp wave signal at half maximum following the peak. The IEI is defined as the distance between the end of an event and the start of the following event. Events in the sharp wave component whose peaks are smaller than 30 pA or separated by <100 ms are discarded from the analysis. As the IEIs are defined based on the FWHM, IEIs <100 ms are possible (see Results), although the peaks are separated by >100 ms. To study the properties of evoked events in Features of spontaneous and evoked SWRs match experimental results, we inject an extra current to randomly selected 60% of *B* cells, at intervals of ∼2 s. For each *B* neuron, the injected current is uniformly sampled from the interval [0, 600] pA and is injected for *T* = 10 ms. To avoid artifacts because of rhythmic stimulation, each stimulation time is shifted by a delay value uniformly sampled from the interval [0, 90] ms. The results of these simulations and those presented in Results do not qualitatively change when current injection to *B* cells is replaced with a depolarizing current injection to *P* cells, or a hyperpolarizing current injection to *A* cells, as long as the stimulation paradigm is strong enough to start a SWR event. Pearson correlation coefficients are computed to estimate the correlation between event amplitude and previous IEI and between event amplitude and next IEI, in both spontaneous and evoked scenarios. Only the properties of evoked events and the interval to the next (or previous) spontaneous SWR events are shown in the analysis of evoked events. The distribution of previous IEI-amplitude pairs is fitted to an exponential function f(x)=a(1−exp(−bx))+c with parameters *a*, *b*, and *c* using a nonlinear least-squares method.

In the simulations with B→P depression and P→A facilitation (shown in Additional short-term plasticity mechanisms), we monitor the system for 10 min and analyze the activity as described above. For the detection of spontaneous events in the scenario where P→A facilitation is the only plastic mechanism, the threshold to detect sharp wave peaks is adjusted to 40 pA and the minimal distance between peaks to 200 ms to account for noisier events.

#### Definition of mean f-I curves in the spiking network

**Figure 4.**
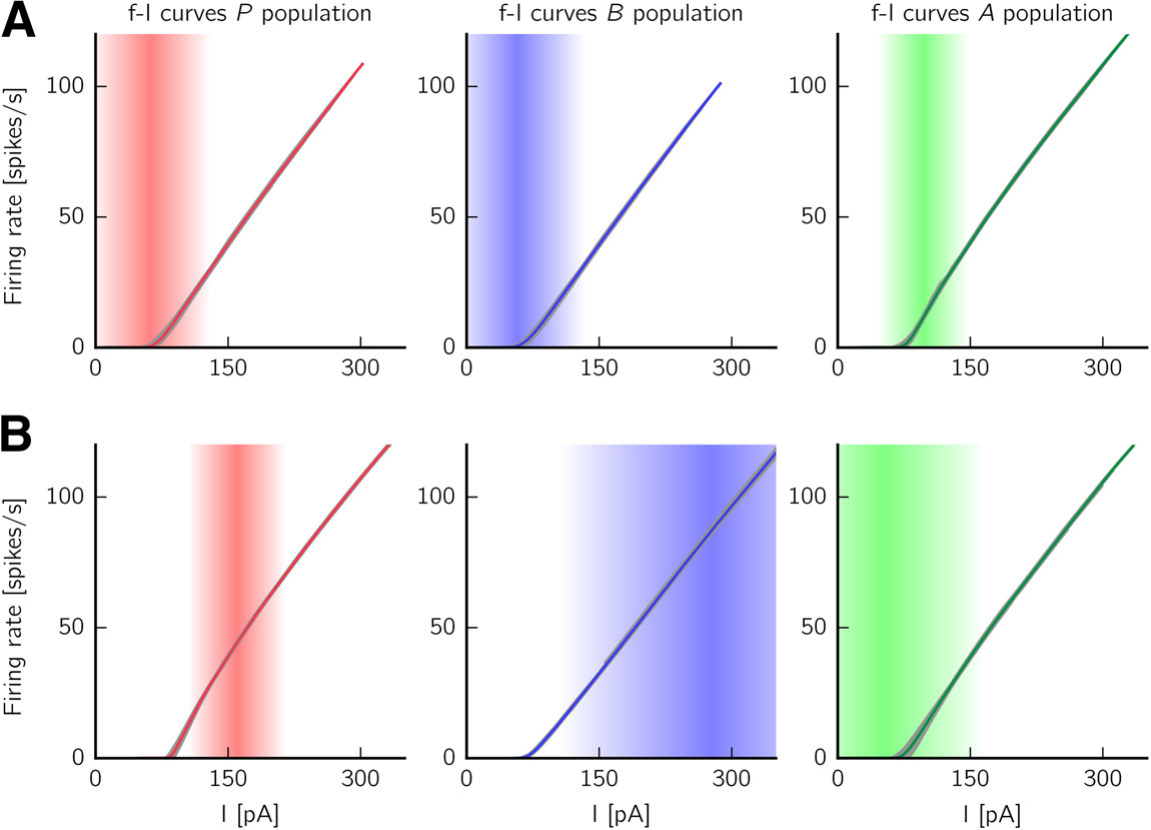
Stationary f-I curves for the bistable spiking network with clamped synaptic efficacy. Displayed are f-I curves of the spiking network as synaptic efficacy is clamped at *e^AB^* = 0.5 (average value of synaptic efficacies of synapses B→A). As shown in Figure 2*A*, a SWR and a non-SWR state coexist in this scenario. Each row indicates the f-I curves for *P*, *B*, and *A* cells (from left to right) in each stationary state (***A***: non-SWR state, top; ***B***: SWR state, bottom). Gray lines indicate the f-I curves of single cells driven by external currents of different intensities. The curves are shifted on the *x* axis to account for average current from incoming synaptic inputs (see Definition of mean f-I curves in the spiking network). Colored solid lines indicate mean curves. Shaded areas represent the regions where most inputs arrive (mean input current ± 1 SD).

To define the spiking neurons' f-I curves shown in Figure 4, we randomly select 50 neurons in each population, and add new neurons to the network with the same neuronal properties and incoming connectivity structure. However, we do not connect these neurons back to the network, i.e., we create copies of the selected neurons in order to study how their activity depends on the input level. To do so, we stimulate these neurons with additional constant currents of different intensities (from −100 to 200 pA, in steps of 5 pA), for *T* = 20 seconds. We distinguish periods during which the network is in either the non-SWR or the SWR state; in the latter case, depolarizing current is transiently injected to the *B* cells in the beginning of the simulation for the system to jump to the SWR state. All neurons in the network also receive a background current of 200 pA, as in all other simulations. We record the mean number of spikes per second, and plot this quantity against the total average input current that the neurons receive. This total current is the sum of the external injected current, the background current, and the synaptic currents caused by incoming presynaptic activity. The gray lines in Fig. 4 depict single neurons' f-I curves. Additionally, the colored lines describe the mean f-I curves, averaged across neurons for each input current value. Finally, to estimate which part of the input range is more relevant to the populations in each state, we define the shaded area. The darker part represents the mean input current value (across time and neurons) seen by a neuron in a given state. The color becomes lighter until it fades at values of mean input ± one SD (value computed by averaging across time and neurons).

### Rate model

#### Motivation

So far, we have introduced a spiking model that reproduces experimental features of SWR generation. As also demonstrated in Results, the spiking model exhibits SWR events spontaneously and in response to current injection, and the SWR dynamics match those seen experimentally. Thus, the spiking model is able, despite its simplicity, to capture the main features of the biological network of interest and to make testable experimental predictions. Additionally, it has the advantage of being defined by variables that are close to experimentally measurable quantities.

However, the large number of parameters makes the system difficult to tune and impedes an understanding of the network dynamics. Why, and for which combination of parameters, does the system reproduce the experimentally observed behavior? What is the impact of one specific parameter on the dynamics of the whole system? How robust is the network to changes of parameters? Answers to such questions remain elusive without a thorough mathematical analysis, that is almost impossible to perform in a spiking network like the one presented above.

This motivates the quest for a simpler network description, where only the average population behavior, and not the single cells' activity, is considered. To this end, we show in what follows how to define a rate approximation of the spiking model. Rate models (Wilson and Cowan, 1972; Breakspear, 2017) provide an accurate representation of the asymptotic behavior of the network under the assumption of describing large and homogeneous populations of neurons (i.e., all neurons share similar intrinsic neuronal properties, receive the same amount of external input, and are coupled by statistically homogeneous connectivity). Similar to Wilson and Cowan (1972), we model the dynamics of the interactions between populations using ordinary differential equations, with an explicit formulation of the populations' input-output transfer functions to allow for the computation of the system's stationary states. The variables *P*, *B*, and *A* describe the average firing rates of the neurons in the three different populations of spiking cells.

#### Rate-model equations

We define the rate model as a set of ordinary differential equations as follows:

(5) $$\begin{array}{ll} \tau_{P}\frac{\partial P}{\partial t} & =-P+f_{P}(W_{PP}P-W_{PB}B-W_{PA}A)\\ \tau_{B}\frac{\partial B}{\partial t} & =-B+f_{B}(W_{BP}P-W_{BB}B-W_{BA}A)\\ \tau_{A}\frac{\partial A}{\partial t} & =-A+f_{A}(W_{AP}P-W_{AB}Be-W_{AA}A)\\ \frac{\partial e}{\partial t} & =\frac{1-e}{\tau_{d}}-\eta_{d}Be, \end{array}$$

where the first three equations describe the dynamics of the populations *P*, *B*, and *A*, and the fourth equation the synaptic depression mechanism, which corresponds to the synaptic depression in the spiking case. *e* modulates the strength of the connection B→A (third equation). The transfer functions (also called activation curves) *f_I_*, with I∈{P,B,A}, describe how a population *I* responds to its incoming inputs. The variables *W_IJ_* are positive and represent the average strength of the synaptic connections from population J∈{P,B,A} to population *I*, and τ*_d_* and η*_d_* are the depression time constant and rate, respectively. In what follows, we briefly sketch how a rate network can be derived starting from the spiking network presented in the previous section.

#### Activation functions

First, we focus on the definition of the activation functions *f_I_*. For asynchronously firing neurons, single neurons' f-I curves are sufficient to define the populations' activation curves (Brunel, 2000; Brunel and Wang, 2003; Gerstner et al., 2014). However, as we have argued in Constructing the spiking network, the spiking network displays bistability for fixed, intermediate values of synaptic depression (see also Figs. 1, 2). For this reason, we need to consider the stationary f-I curves for each population in both SWR and non-SWR states.

In each of the states, the neurons receive a synaptic input that depends on the firing rate of all presynaptically connected neurons in the network. As the firing rates of the populations are drastically different in the two states, we expect the input levels to be also different in either state. To better visualize this effect, Figure 4 shows the mean f-I curves for each population in each stable state (for $e^{AB}=0.5$: average synaptic efficacy in the spiking model). The shaded areas describe the distribution of input currents arriving, on average, to a neuron of a given population in either state; indeed, we can see that they are quite different. Furthermore, the different input levels characteristic of either state also affect the shape of the f-I curves. Indeed, the f-I curve of a neuron receiving noisy inputs from other cells in the network can deviate quite strongly from the f-I curve of the neuron considered in isolation for constant input (Fellous et al., 2003; Gerstner et al., 2014; Shomali et al., 2018).

How can we nevertheless describe a population with a single activation curve? For example, for the *B* population, $f_{B}(I)$ should describe accurately the input-output relation for lower input currents in the non-SWR state (when the synaptic input is I≈57±78 pA, mean ± SD), and for higher input currents in the SWR state (where the input is I≈277±173 pA). Thus, we define an empirical f-I curve by taking the mean f-I curve of the spiking network in the non-SWR state below a given threshold current, and the mean f-I curve of the SWR state above this threshold. The threshold is defined as the current where the mean input current minus 1 SD arrives to the *B* population in the SWR state. This state can be considered as the “active” state for *B* cells because they are almost silent in the non-SWR state.

We then fit this empirical f-I curve to a softplus function f(I)=Fln{1 + exp[k(I + s)]} (Dugas et al., 2001; Glorot et al., 2011) (*F* = 1 spike/s), where the parameters *k* and *s* are optimized via least-square error minimization. The softplus function shows a convex increase for small *I* and grows linearly as k(I+s) for large *I*. For the fitting, *k* [in units of 1/pA] is constrained to the interval [0, 2]; and *s* (in units of pA) is constrained to the interval [–100, 0]. Optimal values for *f_B_* are *k_B_* = 0.41 and *s_B_* = –68.04.

Because the “active” state of the *P* population is the SWR state, the exact same procedure described above applies to the f-I curves of *P*. In the case of the *A* population, whose “active” state is the non-SWR state, the only difference is that the empirical f-I curve is defined by considering the mean f-I curve of the SWR state below threshold (defined as the current value where the mean input current minus 1 SD arrives to the *A* population in the non-SWR state), and the mean f-I curve of the non-SWR state above threshold. Optimal values for the fitted softplus functions *f_P_* and *f_A_* are $k_{P}=0.47,\;s_{P}=-68.34,\;k_{A}=0.48$, and $s_{A}=-68.91$.

To define the three activation functions $f_{P}(I),\;f_{B}(I)$, and $f_{A}(I)$, we additionally include in the input of the rate model the *I_BG_* = 200 pA background current that all neurons in the spiking network receive. In other words, we define $t_{I}=s_{I}+200$ as the threshold of $f_{I}(I)$; no extra background current is injected to the populations in the rate model. Thus, the softplus functions used in the rate-model simulations are as follows:

(6) fI(x)=F ln{1 + exp[kI(x + tI)]}, I∈{P,B,A}

with *F* = 1 spike/s. The parameter values for the rate model are also summarized in Table 5.

#### Time constants

The parameters τ*_P_*, τ*_B_*, and τ*_A_* in Equation 5 set the time constants of the population dynamics. No correspondence can be drawn between the membrane time constants of the spiking network and the population time constants (Abbott, 1994; Dayan and Abbott, 2001; Gerstner et al., 2014). As a result, using the rate model as an approximation of the spiking model can at most hold in the stationary, but not in the transient, case (but for recent approaches that address this problem, see Montbrió et al., 2015; Schwalger et al., 2017). We set the population time constants in Equation 5 to $\tau_{P}=3$ ms, τ*_B_* = 2 ms, and τ*_A_* = 6 ms. These values are biologically plausible (Wilson and Cowan, 1972; Chenkov et al., 2017) and account for the fact that *B* cells are assumed to be fast interneurons; we additionally assume that *A* cells are slower interneurons. However, the asymptotic dynamics is largely independent on the choice of the time constants.

#### Connection strengths

The average strength *W_IJ_* of the connection from population *J* to every neuron in population *I* should depend on the size *N_J_* of the presynaptic population, the connection probability *p^IJ^*, the average unitary conductance increase *g^IJ^* when a presynaptic spike occurs, the average synaptic reversal potential $E_{rev}^{I}$, the average mean membrane potential *V_I_*, and the average conductance decay time constant $\tau_{syn}^{I}$ in the postsynaptic population (Gerstner et al., 2014). More formally, we can define the *W_IJ_* as follows:

(7) $$\begin{array}{ll} W_{IP} & =N_{P}p^{IP}g^{IP}\tau_{syn}^{P}(E_{rev}^{P}-V_{I}),\;I\in \{P,B,A\}\\ W_{IB} & =-N_{B}p^{IB}g^{IB}\tau_{syn}^{B}(E_{rev}^{B}-V_{I}),\;I\in \{P,B,A\}\\ W_{IA} & =-N_{A}p^{IA}g^{IA}\tau_{syn}^{A}(E_{rev}^{A}-V_{I}),\;I\in \{P,B,A\} \end{array}$$

For simplicity, we neglect the synaptic delays in this approximation. The connection strength *W_AB_* is modulated by the synaptic efficacy *e*, which, similarly to the spiking network, is fixed at an intermediate value (in the spiking model: *e^AB^* = 0.5, in the rate model: *e* = 0.5) to ensure bistability. The terms *V_I_* should describe the average membrane potential values of cells in the postsynaptic population *I*. However, in our bistable scenario, the average population membrane potentials differ across the two stable states (because the inputs each cell is receiving change across states). For example, for the *A* population, the mean membrane potential in the non-SWR state is −53.04 ± 2.10 mV (mean ± SD), whereas it is −54.91 ± 1.65 mV in the SWR state. Thus, there is no predetermined way of defining the *V_I_* values. For this reason, we decided to keep *V_P_*, *V_B_*, and *V_A_* as free parameters and run an optimization procedure that searches for values that minimize the distance between the target population firing rates in the spiking model (see Fig. 2*A*) and the population rates of the rate model. More in detail, *V_I_* (I∈{P,B,A}) can range from the reset to the threshold potential. For each possible combination of *V_I_* in this range ([–60, –50] mV, using a step size of 0.5 mV), we run a rate-model simulation for *e* = 0.5 (clamped synaptic efficacy), using the fitted softplus activation functions. The system is initialized to start from the non-SWR state. Current is injected to the *P* and *B* populations (positive current) and to the *A* population (negative current) to let the system jump to the SWR state. We store the population rates in both states if the stimulation is successful, that is, (1) the same two stable states coexist in all three stimulation paradigms; and (2) the firing rate of the stable states are confined to a “biological” range (close to experimental results; Table 4). We note here that most of the combinations of *V_P_*, *V_B_*, and *V_A_* result in rate models with biologically realistic firing rates. Finally, we minimize the Euclidean norm between the vector of target firing rate values in the spiking model and the vector of firing rates in the rate model to find the optimal combination of *V_I_*. In this way, the firing rates in the “active” state of each population (SWR state for *P*, *B*, non-SWR state for *A*) are better matched than the ones for the “inactive” state, which are close to zero. This is a reasonable choice, as the “active” states are the ones that better characterize the firing of a population.

**Table 4.** Summary table for “biological” population firing rates in non-SWR and SWR states

	*P* (s^–1^)	*B* (s^–1^)	*A* (s^–1^)
Non-SWR state	<5	<5	>8
SWR state	>8	>30	<5

**Figure 5.**
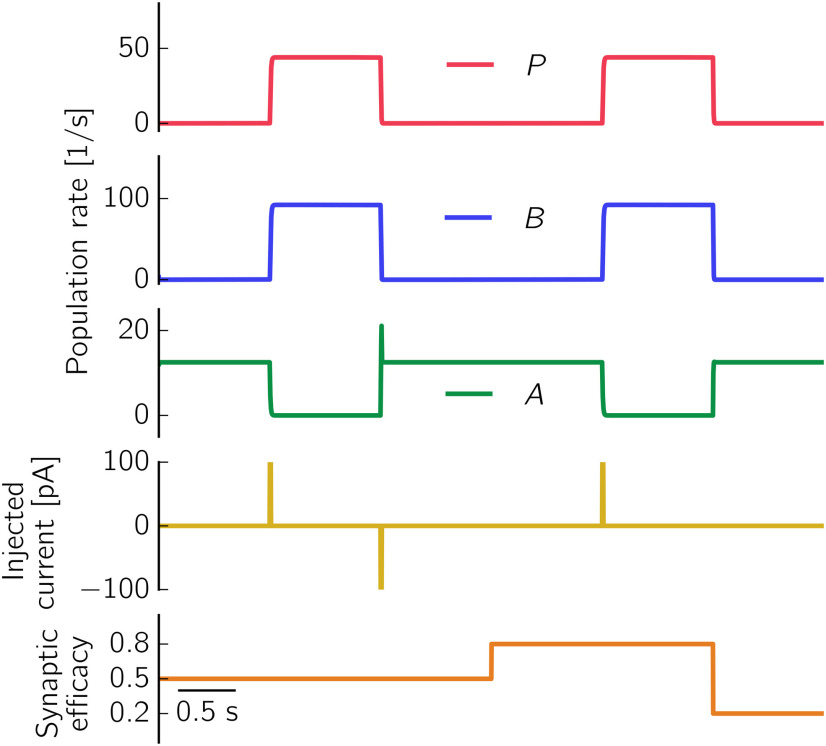
Rate network with fitted softplus f-I curves is bistable for clamped depression values. Top to bottom, Population rates of *P*, *B*, and *A* cells, injected current, and value of synaptic efficacy. When the synaptic efficacy is clamped at *e* = 0.5, two stable states are present in the network (left). Positive current injection to the *P* population (*I* = 100 pA for a duration of 10 ms) triggers the switch to the SWR state, whereas negative current (*I* = –100 pA for a duration of 10 ms) terminates it. These results are comparable to what has been shown in Figure 2*A* for the spiking model. The population firing rate values are matched in both networks because of the optimization of the mean membrane voltages *V_I_* (for details, see Connection strengths). Population rates in the non-SWR state are as follows: *P* = 0 s^−1^, *B* = 0 s^−1^, *A* = 12.5 s^−1^; and in the SWR state as follows: *P* = 44.0 s^−1^, *B* = 92.2 s^−1^, *A* = 0 s^−1^ (for comparison with spiking values, see Fig. 2*A*). Differently from the spiking model, the rate model does not jump to the SWR state for *e* = 0.8 because of its noise-free nature: as the system is fully deterministic, no jumps are expected as far as the change in synaptic efficacy preserves the network bistability. Thus, the rate network is not able to reproduce, in absence of external inputs, fluctuation-driven spontaneous SWRs observed in the spiking network and in experiments. However, when a positive current is injected (so that the system jumps to the SWR state), the event can be terminated by lowering the synaptic efficacy to *e* = 0.2. In this scenario, the *A* population receives too little inhibition from the *B* population and can thus restore its firing rate to non-SWR levels. Network parameters are summarized in Table 5.

For the network configuration presented here, the optimized values are *V_P_* = –52.5 mV, *V_B_* = –54.0 mV, and *V_A_* = –52.5 mV. For *B* and *A*, these values are close to their mean membrane potential values in the “active” state (–54.31 ± 2.94 mV and –53.04 ± 2.10 mV, mean ± SD, for *B* and *A*, respectively). For the *P* population, the optimal value is an average of the peaks of the distributions of membrane potentials in the two states (–54.06 mV and –51.00 mV for non-SWR and SWR state, respectively). This suggests that the optimization yields meaningful results. We use the optimal values of *V_I_* to define the connections *W_IJ_* as described by Equation 7, and use these values to define the rate model used for simulations, an example of which can be seen in Figure 5.

#### Short-term plasticity in the rate model

The last ingredient needed to create the rate model envisioned in Equation 5 is the definition of the synaptic depression equation. It can be directly derived from the spiking case (Eq. 3 with parameters τ*_D_* and η*_D_*) by averaging over realizations (i.e., $e=\bar{e^{AB}}$, where *e^AB^* is the average of the synaptic efficacies $e_{ij}^{AB}$ of synapses j→i, and the bar represents the average over realizations), under the assumption of considering a large number of presynaptic spikes. In this scenario, the synaptic efficacy evolves as described in Equation 5, with $\eta_{d}=\eta_{D}$ and $\tau_{d}=\tau_{D}$.

In Additional short-term plasticity mechanisms, we model a synaptic facilitation mechanism on the P→A connection. We describe the effect of facilitation by multiplying the connection strength *W_AP_* by a factor (1 + *z*), where the variable *z* is described by $\partial z/\partial t=-z/\tau_{f}+\eta_{f}P(z_{max}-z)$. This mechanism is derived from the spiking model (see Eq. 4), with *z* representing the average of *z_ij_* of j→i synapses and over realizations. As done in the spiking model when P→A facilitation is the only short-term plasticity mechanism, we choose $\eta_{f}=0.32,\;\tau_{f}=230$ ms, and *z_max_* = 1.

#### Rate-model noise

To evaluate how well the rate model could capture the transition dynamics between SWR and non-SWR states, we added noise to the current input of the three neuronal populations. Noisy inputs are created to resemble the fluctuations of the spiking model in the non-SWR state, by estimating the currents experienced by a postsynaptic neuron. To obtain noise that resembles the properties of input currents in the spiking model, we separately model the inputs from each of the three presynaptic populations *J* (i.e., *P*, *B*, or *A*) into a postsynaptic neuron (representative of a rate-model population) belonging to population *I* (i.e., *P*, *B*, or *A*). For simplicity, we assume that these nine J→I currents are mutually independent. Each of them is modeled as a homogeneous Poisson process representing the spike times of presynaptic neurons in population *J*. Its frequency is defined by multiplying the spiking network parameters *N_J_* (number of neurons in presynaptic population; Table 1), *p^IJ^* (connection probability for connection J→I; Table 2), and the mean population rate of the presynaptic population in the non-SWR state (see Fig. 2*A*). This spike train is then convolved with an exponentially decaying kernel representing the synaptic current updates; the kernel's time constant is $\tau_{syn}^{J}$ (Table 1), and its amplitude is estimated to be $g^{IJ}\left(E_{rev}^{J}-V_{I}\right)$, where *g^IJ^* is the synaptic conductance increase (Table 2), $E_{rev}^{J}$ is the reversal potential of the presynaptic population (Table 1), and *V_I_* is the estimated mean membrane potential of neurons in the spiking network in the non-SWR state (see Connection strengths). From the noisy input current of a neuron, we subtract the mean because the rate model description of the network already includes the mean currents.

We note that this procedure to generate noise neglects all correlations in the spiking activities, which are considerable in such balanced networks. In order to compensate for this lack of correlations, we heuristically scale down the amplitude of the rate-model noise. We find that scaling down the noise by a factor of 8 allows us to generate SWR events with a similar frequency to those of the spiking model simulations.

In the simulations with additional plasticity mechanisms (in Additional short-term plasticity mechanisms), we perform short simulations of the noisy rate model with extra B→P depression and with P→A facilitation only. For the simulation with extra B→P depression, the noise and rate model parameters are the same as the ones used for the default network (Table 5). For the case with P→A facilitation only, we keep the default rate model parameters but slightly increase the noise amplitude (scaled down by a factor of 7), to be able to trigger spontaneous events.

**Table 5.** Summary of parameters for the rate model*^a^*

Connection strength (pA·s)	*f_I_* slope (1/pA)	*f_I_* threshold (pA)	Time constants (s)
*W_PP_* = 1.72	$k_{P}=0.47$	$t_{P}=131.66$	$\tau_{P}=0.003$
*W_BP_* = 8.86	$k_{B}=0.41$	$t_{B}=131.96$	$\tau_{B}=0.002$
*W_AP_* = 1.72	$k_{A}=0.48$	$t_{A}=131.09$	$\tau_{A}=0.006$
*W_PB_* = 1.24			
*W_BB_* = 3.24	Synaptic depression	Synaptic facilitation	
*W_AB_* = 5.67	$\eta_{d}=0.18$	η_f =_ 0.32	
*W_PA_* = 12.60	$\tau_{d}=0.250$ s	τ_f =_ 0.230 s	
*W_BA_* = 13.44		*z_max_* = 1	
*W_AA_* = 8.40			

*^a^*The synaptic facilitation parameters are used only in the simulations of Figure 15*D*.

#### Quantification of SWR properties in the noisy rate model

To quantify the properties of SWR events in the noisy rate model, we perform 10 min simulations with noise injection (see Rate-model noise), triggering both spontaneous and evoked events, as in the spiking network. To detect events, we apply the script available at Duarte (2015) to a low-pass filtered (up to 10 Hz, which allows for reliable isolation of peaks in the rate model) trace of the *B* population rate. Events whose peaks are <45 s^−1^ or are separated by <100 ms are discarded. We consider a peak's start and end points, from which we calculate the width of an event and the IEI, to be the times at which the half maximum is reached. To evoke events, we inject to the *B* population additional 10 ms square pulses with amplitude 150 pA (sufficient to trigger SWRs, as seen in SWRs can be generated in a CA3-like spiking network) with a periodicity of ∼2 s, with a random additional delay of [0, 90] ms, drawn from a uniform distribution (for a comparison with spiking model simulations, see Quantification of SWR properties).

#### Comparison between spiking and rate model simulations

Now that all the components of the rate model have been defined, we can compare the behavior of the rate model to that of the spiking model presented in Results. Numerical simulations of both models show that there is a qualitative match in the population firing rates (compare, e.g., Figs. 2*A*, 5). Thus, the rate model seems to be a suitable tool to approximate the population dynamics of the spiking model. However, the two models cannot be considered equivalent. First, the rate model is unsuited for describing the transient dynamics of the spiking network, as it can be noted, for example, from the lack of fast (>100 Hz) oscillations in the rate-model simulations (see SWRs can be generated in a CA3-like spiking network). Second, some of the rate-model assumptions are violated: the number of cells in each population is not sufficiently large (as few as 50 cells belong to the *A* population), and the SWR state is not asynchronous (see, e.g., Fig. 3). Third, the process of approximating the spiking network with a rate model is not unequivocal, as it depends on the choice of τ*_P_*, τ*_B_*, and τ*_A_* (population time constants) and *V_P_*, *V_B_*, and *V_A_* (mean membrane potential values used to define the connection strengths *W_IJ_*).

Despite these limitations, the crucial advantage of the rate model over its spiking formulation is that it can be used to predict, as a function of the rate-model parameters, when the network exhibits bistability. In this way, we can understand the influence of each parameter on the behavior of the system and extend the range of bistable solutions to parameters yet untested in the spiking network. The analysis is presented in the next section.

### Bifurcation analysis of rate model

To provide some understanding on the dynamics of the rate model, we used the software XPPAUT (Ermentrout, 2002) to perform a numerical bifurcation analysis. The general aim was to determine how modifying model parameters affected the qualitative model behavior.

#### Key parameters

**Figure 6.**
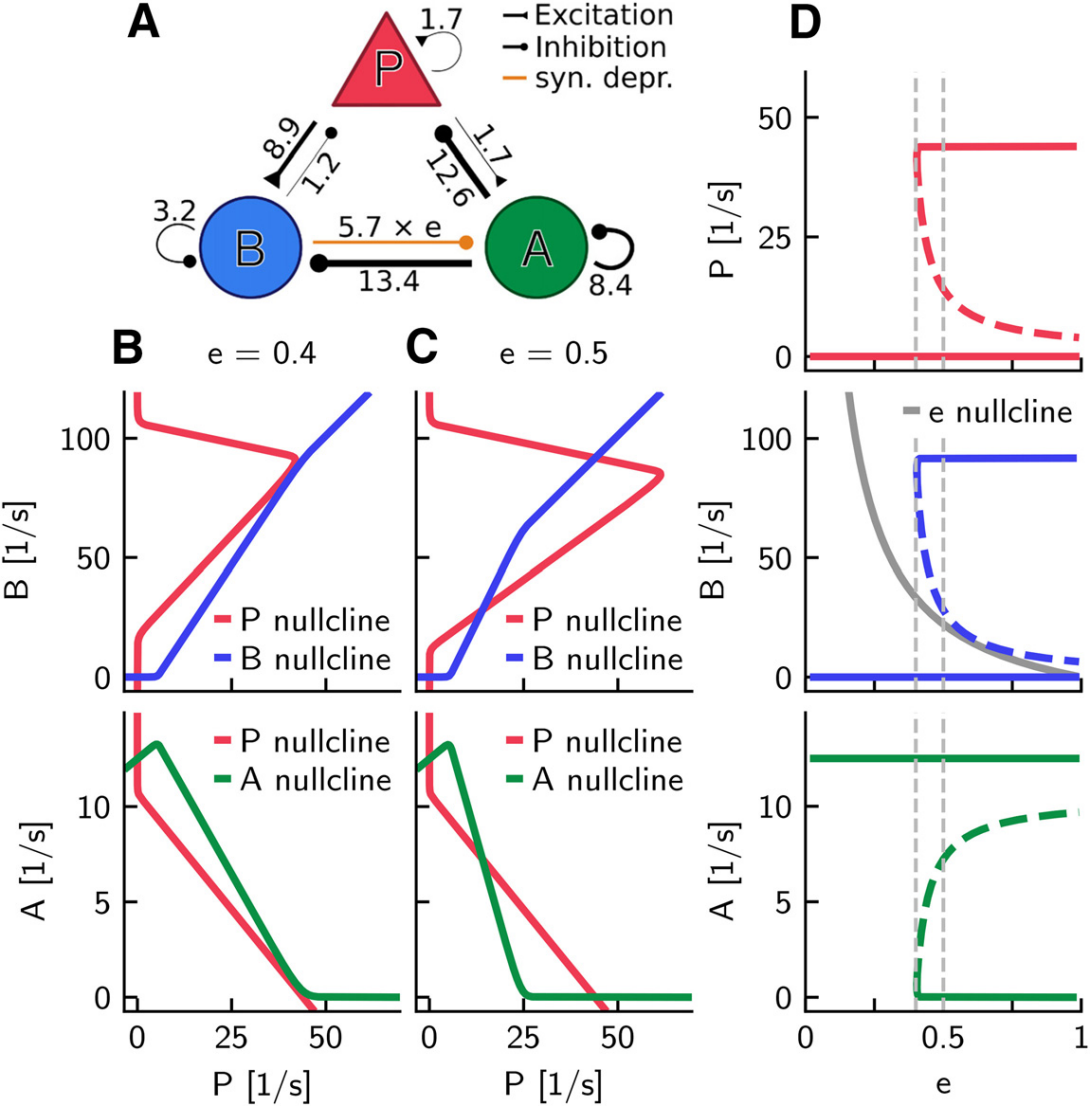
A rate-model approximation of the spiking model reveals its underlying dynamics. ***A***, Circuit with connection strengths, similar to Figure 1*A*. Line width is proportional to the value of the connection strength *W_IJ_* (associated value is near the line) in units of pA · s (for definition, see Eq. 7). ***B***, Nullclines (colored) in the *P*-*B* plane (top) assuming *A* at steady state, and in the *P*-*A* plane (bottom) assuming *B* at steady state for the rate network with softplus activation functions (Eq. 6) and synaptic efficacy *e* = 0.4, just below the bifurcation point *e_crit_* = 0.404 of the connection B→A. Intersections of nullclines are the steady states of the system. ***C***, Same as in ***B***, but for *e* = 0.5. ***D***, Steady-state rates of *P* (top), *B* (middle), and *A* (bottom) as a function of *e*. Solid and dashed colored curves indicate stable and unstable fixed points, respectively. The three bifurcation diagrams show the bistability of the system for $e>e_{crit}$ (coexistence of SWR and non-SWR states). Vertical dashed lines indicate values of *e* in ***B*** and ***C***. Middle, Solid gray curve indicates the *e*-nullcline of the last line in Equation 5. All parameters are summarized in Table 5.

Key parameters of the rate model (Eq. 5) are the connection strengths *W_IJ_* (Eq. 7; default values in Fig. 6*A*). Furthermore, we consider the parameters *k_I_* and *t_I_* of the activation functions *f_I_* in Equation 6. To simplify the analysis, we note that the efficacy *e* is a slow variable. We thus assume that *e* is constant and treat it as another parameter of the model. Because in the bifurcation analysis we evaluate the stability of fixed points of the dynamics, the time constants τ*_I_* can be neglected.

#### Nullclines and fixed points

The method for obtaining fixed points is illustrated in Figure 6*B* in which the efficacy *e* is set to 0.4. The top panel (*P*-*B* plane) shows the *P*- and *B*-nullclines assuming *A* at steady state. The intersection of the nullclines at *P* = *B* = 0 indicates the steady state of the system. In the *A*-*P* plane (bottom, assuming *B* at steady state), the *P*- and *A*-nullclines intersect at *P* = 0 and *A* = 12.5 spikes/s. Together, for *e* = 0.4, there is only one fixed point of the system. In Figure 6*C*, we considered a slightly higher efficacy (i.e., *e* = 0.5). In this case, the intersections of the nullclines show the existence of three steady states, indicating a qualitative change of the dynamics as a function of *e*.

#### Bifurcation diagrams

The dependence of the steady-state rates of *P*, *B*, and *A* on *e* as well as the stability of these fixed points are summarized in Figure 6*D*, which reveals the existence of a bifurcation at the critical value *e_crit_*. For $e<e_{crit}$, there is only a single fixed point, which we associate with the non-SWR state (*P* = *B* = 0, *A* > 0). On the other hand, for $e>e_{crit}$, the network is bistable: there is an additional stable state in which *P* and *B* have positive firing rates but *A* = 0, which we associate with the SWR state. The unstable branch (Fig. 6*D*, dashed lines) can be interpreted as a threshold for transitions between the two stable states. The threshold is closer to the non-SWR state for larger *e* values, which suggests that a smaller perturbation (or favorable stochastic fluctuation in a corresponding spiking model) can evoke a transition to a SWR state.

#### Fast-slow analysis

Figure 6*D* allows also a “fast-slow” interpretation of the dynamics of SWRs. So far, we have assumed that *e* is a slow variable, and treated it as a parameter in the rate model (see Eq. 5), but the efficacy *e* does change, and the change is different in the SWR state and the non-SWR state. To see how *e* drifts, we added in Figure 6*D* the *e*-nullcline (solid gray curve), which is in between the middle (threshold) branch and above the lower branch (non-SWR state) for $e>e_{crit}$; thus, *e* is increasing in the non-SWR state and decreasing in SWR state.

When the system is initialized in the SWR state, a slowly decreasing *e* leads to a transition to the non-SWR state at *e_crit_*. The time needed to reach the transition point explains the duration of a SWR. In the non-SWR state, *e* increases, and the time needed until a fluctuation can induce a transition to the SWR state determines the interval between SWRs. Because we have attributed the change of *e* to a *B*-dependent synaptic depression mechanism, the speed of decrease of *e* is determined by the firing rate of *B* during the SWR state and the depression parameter η*_d_* = 0.18; in contrast, the speed of increase is determined only by the time constant τ*_d_* = 250 ms of recovery from depression (Eq. 5). This distinction enables SWRs to have durations much shorter than the intervals between successive SWRs. Furthermore, the need for a recovery of *e* predicts some refractoriness after a SWR. The network can therefore be classified, according to the terminology in Levenstein et al. (2019), as being in an excitableDOWN regimen.

#### Dependence of bistability on weights

**Figure 7.**
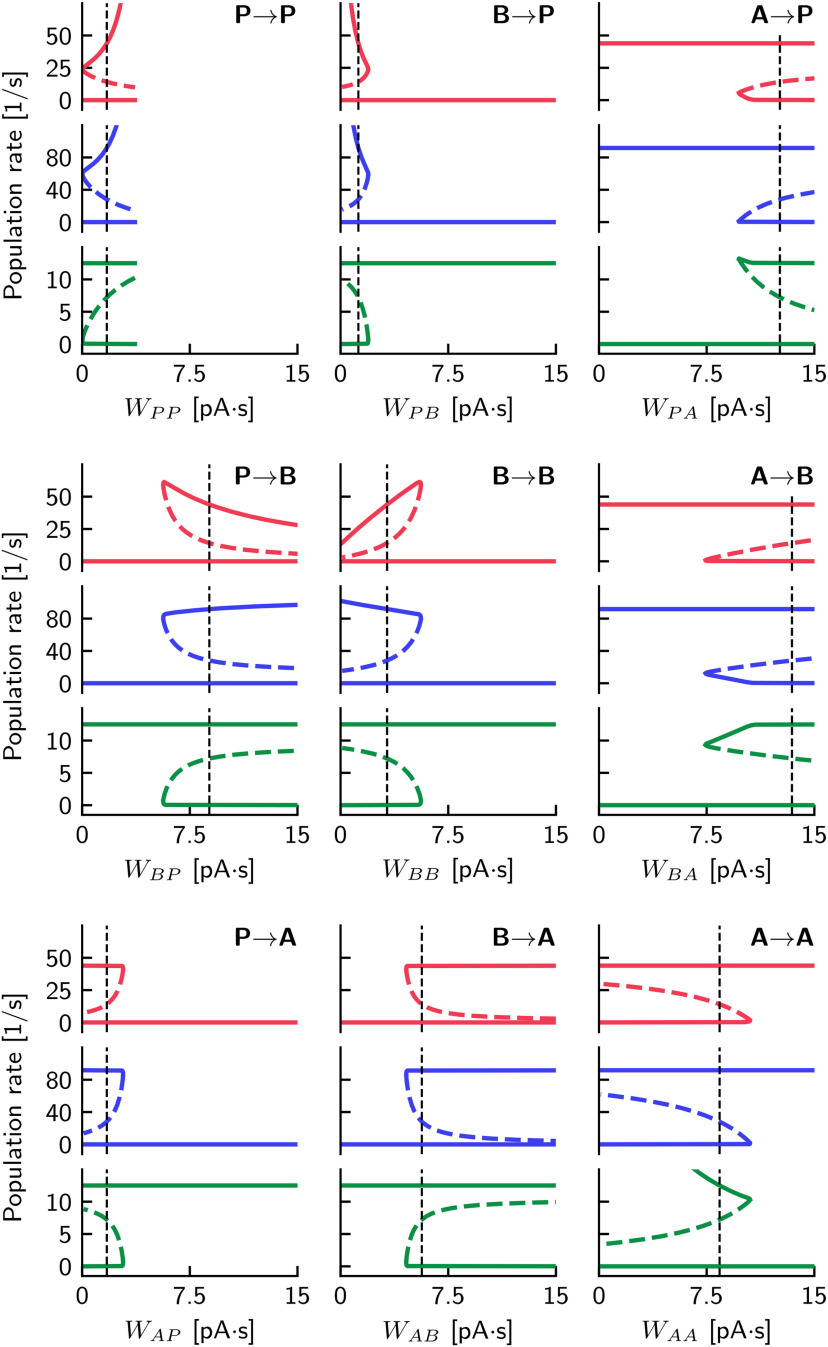
Bistability in dependence on connection strengths reveals the robustness of the model. Steady-state rates of *P* (top), *B* (middle), and *A* (bottom), for each of the nine connection strengths *W_IJ_* in the rate model. All weights are varied from 0 to 15 pA · s. Solid and dashed colored curves indicate stable and unstable fixed points, respectively. Vertical dashed lines indicate the default values stated in Figure 6*A*. For all calculations, we have fixed *e* = 0.5. Further parameters are summarized in Table 5. Since the upper stable branches of *P* and *B* in the P→P connection grow to infinitely high values for a large weight, we show *W_PP_* only in the range from 0 to 3.7 pA · s, above which the numerical continuation of the steady state cannot be made. Except for *W_PP_*, all weights have a critical value separating monostable and bistable regions.

The particular type of bistability of the network is thus a key feature of the rate network, and in Figure 6 we have investigated this property as a function of the efficacy *e*. Figure 7 extends this analysis and illustrates the dependence of fixed points on the nine connection strengths (for the efficacy fixed to *e* = 0.5). The nine panels in Figure 7 are similar in structure to Figure 6*D*, which is partly identical to the panel for the connection from B→A (weight *W_AB_*) because this connection strength is equal to the product $e\cdot W_{AB}$. This panel is also similar to all other panels (except the one for *W_PP_*) in that there exists a critical weight that separates bistable and monostable regions.

For large *W_PP_*, the *P* and *B* firing rates in the SWR state can reach infinitely high values. Indeed, we found numerical continuation of this steady state to be impossible for *W_PP_* > 3.8 pA · s. Although the non-SWR steady state remains unchanged in this region, any small perturbation that brings the system over the threshold would lead to an unbounded growth in *P* and *B*. For this reason, in Figure 7, we only show these steady states in the region *W_PP_* < 3.8 pA · s.

#### Robustness of the model

**Figure 8.**
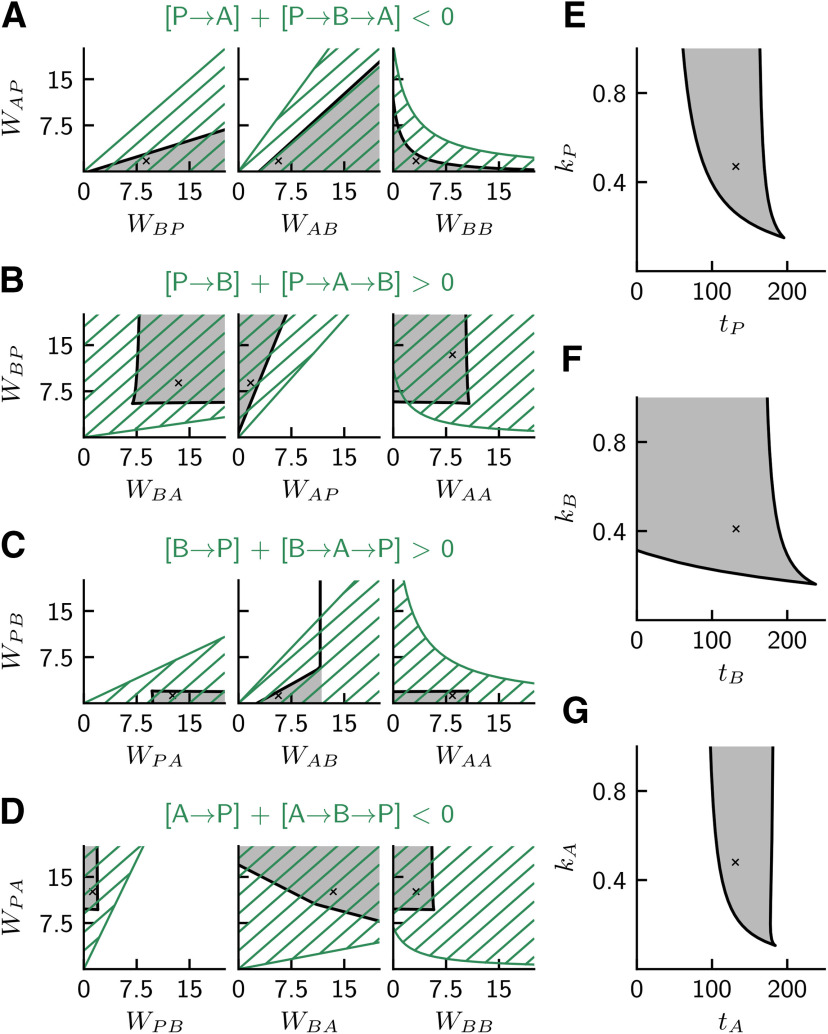
Bistable regions in two-dimensional slices of parameter space. Each plot represents the network behavior with respect to a pair of parameters of the rate model. Black lines indicate a numerical continuation of the bifurcation point separating the bistable and monostable regions along two model parameters. Dark gray areas represent the bistable region where both SWR and non-SWR states coexist. Black crosses represent the standard chosen parameter values for the rate model (summarized in Table 5). ***A-D***, Bistability with respect to connection strengths *W_IJ_* (in units pA · s) that contribute to the four pathway-strength requirements. For comparison, the hatched green areas represent the region where the requirements are met in their linear approximation (see Pathway strengths and quantification of requirements). ***E-G***, Bistability with respect to slopes *k_I_* (in units 1/pA) and thresholds *t_I_* (in units pA) of the softplus activation functions (Eq. 6) for *P*, *B*, and *A*.

Figure 7 highlights the robustness of the rate model: for each weight, there is a wide range of values in which the system is bistable. Moreover, the firing rates (i.e., the values of stable fixed points in the bistable regimen) are constant for large ranges of some weights. To intuitively understand this feature, let us first focus on the SWR state, which was defined to have *A* = 0, *P* > 0, and *B* > 0. Because of *A* = 0, the rates of *P* and *B* are independent of the weights of the three connections emerging from *A* (i.e., *W_PA_*, *W_BA_*, and *W_AA_*; Fig. 7, right column). Moreover, the values of the firing rates of *P* and *B* are independent of *W_AB_* if this inhibition is beyond its critical value such that *A* is silenced. *P* and *B* are also independent of *W_AP_* if this excitation is below its critical value so that *A* is not active. The other four weights, which involve the connections to and from the *P* and *B* populations (i.e., *W_PP_*, *W_BP_*, *W_PB_*, and *W_BB_*) could be used to regulate the desired values of firing rates of *P* and *B* in the SWR state. Similar arguments supporting the robustness of the rate model hold for the non-SWR state, which was defined to have *P* = *B* = 0 and *A* > 0. Because of *P* = *B* = 0, the rate of *A* is independent of the weights of the six connections emerging from *P* and *B* (i.e., *W_PP_*, *W_BP_*, *W_AP_*; *W_PB_*, *W_BB_*, *W_AB_*; Fig. 7, first and second columns). The three inhibitory connections emerging from *A* constitute a special case: the value of *W_PA_* is irrelevant only if it is large enough (above some threshold) so that *P* = 0. Similarly, *W_BA_* is uncritical if it is large enough such that *B* = 0. The recurrent weight *W_AA_* (if below some critical value) can be used to set the firing rate of *A* > 0, which involves, however, an additional excitatory input (parameter *t_A_* in our model, see also Fig. 8).

#### Essential connections and minimal network

Figure 7 helps to identify essential connections in the rate network. For example, *W_AP_* is not critical, that is, the system is bistable as long as *W_AP_* is sufficiently small, and it can be even set to zero. Furthermore, *W_PB_* and all recurrent connections (*W_AA_*, *W_BB_*, *W_PP_*) should be weak enough, and could, in principle, be eliminated from the rate network without changing its qualitative behavior.

These observations raise the question regarding the identity of the minimal circuit that would support bistability in the rate model. Further simulations in which we simultaneously varied several weights confirmed that it is indeed possible to set $W_{AP}=W_{PB}=W_{BB}=W_{PP}=W_{AA}=0$ and retain bistability, that is, two stable steady states separated by a threshold (the unstable steady state). However, it is not enough for the system to be simply bistable; rather, we want it to allow for transitions between both stable states, corresponding to the initiation and termination of SWR events (as explained in Fast-slow analysis). For a small perturbation to be able to carry the system from the non-SWR to the SWR state, we require that the distance between the threshold and non-SWR branch is relatively small. For $W_{AP}=W_{PB}=W_{BB}=W_{PP}=0$, we found that decreasing *W_AA_* had the effect of bringing the threshold farther and farther away from the non-SWR state (the same happens on the default network, as seen in Fig. 7, A→A), requiring a larger and larger perturbation to trigger a SWR event; for *W_AA_* = 0 it became virtually impossible to start an event. Furthermore, we found that it was necessary to decrease the value of *W_AB_* to terminate an event through B→A synaptic depression in the minimal network. Setting $W_{AP}=W_{PB}=W_{BB}=W_{PP}=0$ brought the value of *e_crit_* (see Fig. 6*D*) so close to zero that transition from the SWR to the non-SWR branch could never happen. By decreasing the value of this connection strength (e.g., *W_AB_* = 4 pA · s), we were able to increase *e_crit_* enough to recover the ability of the depression mechanism to terminate the event (to see how *W_AB_* directly influences *e_crit_*, compare Fig. 6 with Fig. 7, B→A).

#### Dependence of the threshold on parameters

A favorable condition for evoking a SWR by weak stimulation (or by a fluctuation in the spiking model) is one in which the threshold (the unstable branch) is close to the stable branch of the non-SWR state; that is, the distance between stable and unstable branches is small. So, how does this distance depend on parameters? The bifurcation diagrams in Figure 7 indicate that, in addition to *W_AA_*, which we have discussed in the previous paragraph, many other weights can regulate the threshold. Let us classify the weights according to their values at which the threshold is closest to the non-SWR state. First, the three weights emerging from *A* (i.e., *W_PA_*, *W_BA_*, *W_AA_*) show that the distance between the stable (non-SWR state) and unstable branches is the smallest close to the bifurcation point; thus, those weights are not suited for a robust regulation of the threshold. Second, for the three “nonessential” weights *W_PB_*, *W_BB_*, and *W_AP_*, the distance between threshold and the non-SWR stable branch is smallest at zero weight, which further indicates their minor importance in this model. Third, the three weights *W_PP_*, *W_BP_*, and *W_AB_* give rise to smaller distances between threshold and the non-SWR branch for larger weights. *W_PP_* is special because the network becomes unstable for recurrent excitation which is too large (“empty space”, Fig. 7, top left). The most interesting candidates for a (dynamic) control of the threshold are *W_BP_* and *W_AB_*. In what follows, we focus on *W_AB_* because this connection can be dynamically regulated by synaptic short-term depression, which we have described by the efficacy *e*. In contrast, experimental evidence indicates that the connection *W_BP_* is governed by facilitation (Nanou et al., 2018) and thus cannot give rise to the envisioned fast-slow dynamics of SWRs in the proposed setup.

To understand how the threshold depends on *W_AB_* in Figure 7, we note that, even for large weight values (equivalent to *e* > 1 in Fig. 6*D*), the threshold state is very close to (but does not merge with) the stable non-SWR state: for larger *W_AB_* the impact of *B* on *A* is stronger, but only if *B* > 0. In contrast, for *B* = 0 the impact on *A* is independent of *W_AB_*. Thus, the threshold remains always at firing rates *B* > 0. We again note that the proximity of the (unstable) threshold branch and the stable non-SWR is key for eliciting SWRs by small current pulses (and fluctuation-induced SWRs in the spiking model).

#### Predictions derived from the rate model

Finally, we summarize the predictions (obtained from Figs. 6, 7) about the properties of the proposed population *A* of interneurons: Large enough inhibitory connections A→P and A→B are required; the strength of the connections should ideally be well above some critical values. Moreover, the existence of a sufficiently strong inhibitory connection from B→A is required (the larger the connection strength, the smaller the threshold). The interpretation of these constraints is twofold: (1) mutual inhibition between *B* and *A* is key for bistability; and (2) the connection A→P mediates disinhibition (together with a strong enough pathway B→A→P). These predictions are in line with the four requirements on pathway strengths that we postulated in Requirements on pathway strengths.

#### Two-parameter bifurcation analysis and parameter sensitivity

To further illustrate the robustness of the rate model with respect to parameter values, we show two-parameter bifurcation diagrams in Figure 8. The dark gray regions depict the range of bistability with default parameter values marked by crosses. Figure 8*A–D* shows various combinations of pairs of weights *W_IJ_*, and Figure 8*E–G* shows pairs of threshold (*t_I_*) and slope (*k_I_*) parameters of the activation functions. Parameter sensitivity is indicated by wide ranges (i.e., large gray areas) of combinations of pairs of connection strengths (Fig. 8*A–D*), which extends the insights obtained from varying single weights in Figure 6. There is also a wide range of parameter combinations of thresholds and slopes allowed (Fig. 8*E–G*). To evaluate, for example, the requirements regarding the properties of population *A*, Figure 8*G* shows that *t_A_* should be large enough so that *A* is spontaneously active in non-SWR state, and that the particular value of $k_{A}>0$ is uncritical.

#### Pathway strengths and quantification of requirements

The particular combinations of weights and their arrangement in the four rows Figure 8*A–D* are chosen for comparison of the rate model with the four requirements for pathway strengths that were elaborated for the spiking model. For example, Figure 8*A* has the title [P→A]+[P→B→A] < 0, which summarizes our requirement 1: “pathway P→B→A should be stronger than P→A ”, which guarantees that the activation of *P* decreases *A* (expressed by “< 0” in the inequality). We thus need to consider here that the strength of the excitatory pathway [P→A] is positive and that of the inhibitory pathway [P→B→A] is negative. Major players for the strengths of the two compared pathways are the weights displayed in Figure 8*A*.

To enable a quantitative comparison between the prediction of a requirement and the results of a (numerical) bifurcation analysis in Figure 8*A–D*, we formally define the pathway strength as the partial derivative $\partial I/\partial J{|}_{K}$ where *J* is the rate of the presynaptic population, *I* is the rate of the postsynaptic population, and *K* is the (constant) rate of the “third” population. We then use the definition of the rate model in Equation 5 in steady state and the definition of the activation function in Equation 6 in its linear approximation (i.e., $f_{I}(x)=Fk_{I}(x+t_{I})$ with *F* = 1 spike/s); the latter considerably simplifies the approach and enables an explicit formulation of the pathway strengths (which would otherwise depend on the state). For example, the strength of the direct pathway P→A is then as follows:

(8) $${\frac{\partial A}{\partial P}|}_{B}=\frac{k_{A}\;W_{AP}}{1-k_{A}\;(-W_{AA})}\;.$$

Moreover, an indirect pathway I→J→K (i.e., from *I* to *K* via *J*) can be defined as the product of strengths of the pathways I→J and J→K. For example, the strength of the indirect pathway P→B→A is as follows:

(9) $${\frac{\partial B}{\partial P}|}_{A}\cdot \;{\frac{\partial A}{\partial B}|}_{P}=\frac{k_{B}\;W_{BP}}{1-k_{B}\;(-W_{BB})}\cdot \frac{k_{A}\;(-W_{AB})}{1-k_{A}\;(-W_{AA})}\;.$$

We define *W_IJ_* > 0 for both excitatory and inhibitory weights; moreover, the fact that *A* and *B* are inhibitory enters the equation via minus signs in front of *W_AA_*, *W_AB_*, and *W_BB_*. We thus can derive inequalities from the four requirements, which are visualized in Figure 8 (green hatched regions). For example, requirement 1 in Figure 8*A* can be expressed as follows:

(10) $$W_{AP}\cdot (1+k_{B}\;W_{BB})<k_{B}\;W_{BP}\;W_{AB}\;.$$

The overlap of hatched and gray regions in Figure 8 indicates a good match between the requirements derived from the (linear) approximation of the pathway strengths and the numerical bifurcation analysis of the (nonlinear) rate model, which supports the intuition about the network behavior that we have developed earlier.

#### Statistical analysis

To test the existence of a linear correlation between SWR amplitude and length of previous and next IEIs, we have used the Pearson correlation coefficient and reported the corresponding *p* values in Results. Data are mean ± SD.

#### Code accessibility

Sample code to reproduce all figures of this manuscript is available in the GitHub repository (https://github.com/robyeva/SWR_generation_disinhibition).

## Results

To test the hypothesis of disinhibition-mediated generation of SWR events, we consider a computational model comprising a population of pyramidal cells (*P* in the model) and two populations of different types of interneurons: PV^+^ BCs (*B* in the model) and a class of yet unidentified anti-SWR cells (*A* in the model). Neurons are recurrently connected as depicted in Figure 1*A*.

As SWRs have been shown to originate in isolated CA3 slices (Maier et al., 2003; Nimmrich et al., 2005), we constrain the model to reproduce the basic features of the CA3 microcircuit in a rodent slice. Details of the approach are outlined in Materials and Methods. Briefly, we include 8200 *P* (pyramidal cells) and 135 *B* cells (PV^+^ BCs). Additionally, we include 50 *A* cells (anti-SWR cells) in the network. Neurons are randomly connected with connection probabilities that have been chosen to reproduce, if available, experimentally verified connectivity in CA3. The proposed connections from and to anti-SWR cells are such that the population firing activities of *P* and *B* cells in non-SWR periods and during SWR events are close to experimental values.

As demonstrated below (and in Materials and Methods), the model is governed by disinhibition: when *B* cells are activated, tonically active *A* cells are inhibited, and thus release the *P* cells from inhibition. This pattern of activity (increased firing rate of *P* and *B* cells, decreased firing of *A* cells) is characteristic of a SWR event. A SWR terminates when the high activity of *A* cells is restored, and the activity of *P* and *B* cells is low.

The disinhibition mechanism suggests that the two different interneuron populations could be active at different stages: in the non-SWR state, highly active *A* cells control the firing of *P* cells and *B* cells, which are almost inactive. In the SWR state, increased activity of *B* cells inhibits *A* cells; thus, *B* cells are the dominant interneuron class controlling the firing of *P* cells during SWRs. The competition between the interneurons is the mechanism that effectively regulates the alternation between SWR and non-SWR states (Fig. 1*B*).

To construct the network by adjusting synaptic conductances within and across homogeneous populations, we assume for simplicity that the inactive interneuron population in either state is silent, and focus on the two subnetworks *P*-*A* and *P*-*B*. Once the connections are chosen for the subnetworks to represent the non-SWR and SWR states, respectively, we create the full network by adding the reciprocal connections between *B* and *A* cells. The inhibitory connection B→A is equipped with a short-term synaptic depression mechanism, which is progressively reducing the efficacy of the B→A synapses as the firing of *B* cells is high. Thus, depression provides a mechanism to terminate SWRs by releasing the inhibition of *A* cells in SWR states, when *B* cells are highly active. All the other connections are static (but see Additional short-term plasticity mechanisms). The existence of synaptic depression at the B→A synapses is inspired by the results of Kohus et al. (2016), who showed that a PV^+^ BC-mediated depression mechanism could influence the timing between successive SWR events *in vitro*. The details on how to construct the network are provided in Materials and Methods. All model parameters are listed in Tables 1–3.

### The spiking network as a perturbed bistable system

To illustrate basic features of the spiking network, we first discuss simulations in which the synaptic depression is artificially fixed. In this way, we show that the network can be described as a bistable system.

Figure 2*A* shows that the system switches between non-SWR and SWR states on current injection; there the synaptic efficacy variables $e_{ij}^{AB}$ (regulating the depression strength of the synapse between neuron *j* in population *B* to neuron *i* in population *A*; see Eq. 3) are artificially clamped at an intermediate value of $e_{ij}^{AB}=0.5$ for all B→A synapses, so that the average synaptic efficacy is $e^{AB}=0.5$. The SWR state is initiated via a depolarizing-current injection to the *P* cells (60% of cells are stimulated, mean injected current is $I_{\text{mean}}=150$ pA for a period of 10 ms; see Materials and Methods). The SWR state is characterized by high activity of *P* and *B* cells (*P* ≈ 43 spikes/s and *B* ≈ 92 spikes/s) and almost silent *A* cells (*A* ≈ 1 spike/s). This state is sustained until a hyperpolarizing current is applied to *P* cells ($I_{\text{mean}}=-150$ pA on average injected to 60% of cells), which brings the system back to the non-SWR state. The non-SWR state is characterized by intermediate and low activity of *P* and *B* cells (*P* ≈ 2 spikes/s and *B* ≈ 1 spike/s), and tonic activity of *A* cells (*A* ≈ 12 spikes/s). Thus, the network is in a bistable configuration, in which two stable states (non-SWR and SWR states) can be observed. As depicted in Figure 2*B*, each of the two states is characterized by a dominant subnetwork formed by the pyramidal cells and the active interneuron type (*A* and *B* cells in non-SWR and SWR states, respectively). External stimulation (in this example, current injection to *P* cells) can induce transitions between the two states. A transition can also be induced by varying the synaptic efficacy (without current injection). For example, increasing the synaptic efficacy to large values (from *e^AB^* = 0.5 to *e^AB^* = 0.8 in Fig. 2*A*) can result in a jump to a (long-lasting) SWR state. The transition to the SWR state is induced by intrinsic fluctuations present in the network, caused by the random connectivity and the finite size of the network. We observed a small delay (∼25 ms) between the clamping of the synaptic efficacy to *e^AB^* = 0.8 (Fig. 2*A*, right dashed line) and the start of the SWR state; the length of the delay decreases with increasing synaptic efficacy value and strength of fluctuations, and the delay varies across trials (not shown) because noise kicks the network from the non-SWR to the SWR state. The value of the population firing rates in the SWR states depends weakly on the value at which the synaptic efficacy is clamped, and the level of fluctuations changes for different *e^AB^* values (compare SWR states in Fig. 2*A*). Finally, Figure 2*A* shows that, when the synaptic efficacy is decreased to *e^AB^* = 0.2, the non-SWR state is restored, and the system rests in this state in absence of further network modifications.

To better understand the bistable nature of the network, we approximated the spiking model to a rate model, in which only the average population activity of *P*, *B*, and *A* cells, and not the behavior of each single neuron, is considered. The steps required to reduce the spiking model to a rate-model formulation are presented in Rate model. The key advantage of the rate model is that it allows to explicitly study the existence of bistability as a function of few essential network parameters. For example, in Figure 6*D*, we use bifurcation analysis to show that the existence of bistability critically depends on the value of *e* (*e^AB^* in the spiking model). This analysis thus supports the results presented in Figure 2*A* for the spiking model. Additionally, in Figures 7 and 8, we summarize how bistability depends on the strength of mutual connections among populations and on their input-output functions. These results show that the bistable configuration is a general property of such networks with fixed synaptic depression (constant *e*, *e^AB^*) and is thus robust to changes in model parameters.

A synaptic depression mechanism that is not fixed, but can evolve dynamically (see Eq. 3), can be tuned to induce the termination of the SWR states after 50-100 ms. This mechanism assures that SWRs emerge in the network as brief perturbations of the default non-SWR state, as shown below.

### SWRs can be generated in a CA3-like spiking network

#### Spontaneous SWRs

**Figure 9.**
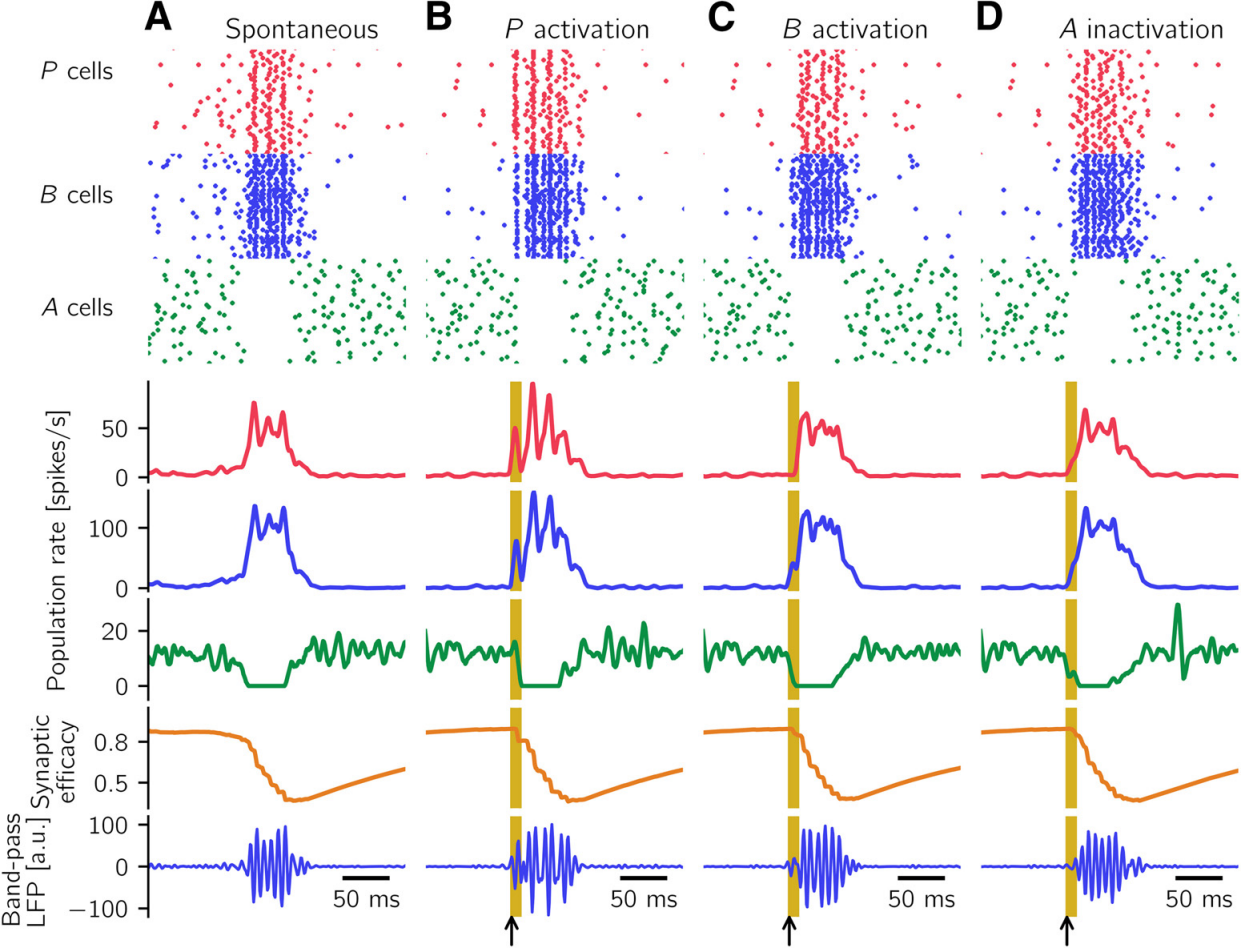
SWR events can be generated in the spiking network. Each column describes one simulation. ***A***, Spontaneous SWR events can be generated in the network. Displayed are raster plots for 50 cells of the *P*, *B*, and *A* populations (red, blue, and green, respectively; each row is one neuron), averaged population firing rates, the time course of the synaptic depression mechanism, and the bandpass filtered (90-180 Hz) component of the LFP signal (see Materials and Methods and Fig. 3). ***B***, ***C***, A fraction of all *P* and *B* cells are stimulated with depolarizing current for 10 ms (yellow areas, black arrows). The stimulation elicits SWR events comparable with the spontaneous ones, in agreement with experimental results. ***D***, A fraction of *A* cells is stimulated with hyperpolarizing current for 10 ms. The stimulation elicits SWR events comparable with the spontaneous ones; this is a prediction of the model. Parameters used to simulate the spiking network are listed in Tables 1–3.

*In vitro*, SWRs occur spontaneously in CA3 (Maier et al., 2003; Ellender et al., 2010). In Figure 9*A*, we show that a spiking network with dynamically evolving B→A depression produces a spontaneously occurring SWR event. The network has been initialized to start in the non-SWR state, characterized by low activity of *P* and *B* cells, and tonic activity of *A* cells (same as non-SWR state in Fig. 2). In this setup, the SWR state is characterized by high activity of *P* and *B* cells and almost silent *A* cells (corresponds to SWR state in Fig. 2). The initiation of a SWR event is triggered by the intrinsic fluctuations present in the network (no current is injected). For example, an increase in the firing of *B* cells decreases the activity of *A* cells, which in turn increases firing of *P* cells, which increases the activity of *B* cells and could even overrule the inhibition from *A* to *B* cells. When the activity of *B* cells is high, the synaptic efficacies of the connection B→A decrease (orange trace in Fig. 9 is the mean synaptic efficacy *e^AB^*). As a result, the *A* cells are progressively less inhibited by *B* cells and thus increase their firing and, consequently, their inhibitory drive onto *P* cells. Thus, the depression at the B→A synapses terminates the SWR event, and the population firing rates are reset to their non-SWR values. The orange trace in Figure 9*A* shows that the synaptic efficacy is restored more slowly than the population activities. We note that the value $e^{AB}=0.5$ is linked to both the SWR state (descending part of the synaptic efficacy trace) and the non-SWR state (ascending part of the trace), confirming the existence of bistability for intermediate values of synaptic efficacy (see also the bifurcation analysis of the rate model in Fig. 6).

To be able to better compare our results to experimental SWRs, which are usually recorded as the LFP of stratum pyramidale, we need to define a measure for the LFP in our setting. The exact origin and cellular contribution to the LFP remain elusive, and the subject is a matter of intense debate (see, e.g., Einevoll et al., 2013). We decided to approximate the stratum pyramidale LFP as a filtered version of the average input current from *B* cells to *P* cells (Beyeler et al., 2013; Schönberger et al., 2014) (see Fig. 3; and Defining the LFP). Interestingly, the last row of Figure 9*A* shows that the bandpass filtered (90-180 Hz) LFP displays ripple-like oscillations with a peak frequency of 135 Hz (peak of the power spectrum). However, we note that our model, which focuses on describing the dynamics of sharp waves, is not intended to predict the frequency of ripple oscillations. The ripple frequency may depend on axonal delays (Brunel and Wang, 2003; Donoso et al., 2018), which we have fixed here to 1 ms for simplicity.

#### Stimulation-induced SWRs

In addition to spontaneous SWRs, Ellender et al. (2010), Schlingloff et al. (2014), Stark et al. (2014), Bazelot et al. (2016), and Kohus et al. (2016) have shown that SWR events can be triggered by cell stimulation. The model can reproduce these results. Figure 9*B* shows that injecting a depolarizing current pulse (*I*_mean_ = 150 pA; see Materials and Methods) to a fraction (60%) of *P* cells results in a SWR event (in line with experiments by Stark et al., 2014; Bazelot et al., 2016). This induced SWR event is qualitatively similar to the spontaneous SWR event in Figure 9*A*. In addition, activating *B* cells (Fig. 9*C*) by injecting a depolarizing current (*I*_mean_ = 250 pA) to a fraction (60%) of cells results in a SWR event, comparable to the experiments reported by Schlingloff et al. (2014) and Kohus et al. (2016). Finally, the model predicts that injecting a hyperpolarizing current to the *A* cells (*I*_mean_ = –250 pA injected to 60% of the cells) results in the generation of a SWR event. Overall, the features of SWRs displayed in Figure 9*B–D* are comparable, although current is injected to different groups of cells.

The results of current stimulation presented in Figure 9 crucially depend on the average connectivity in the network: if, for example, the connection B→P is too strong, activating *B* cells could result in a decrease of firing in *P* cells, and not in a disinhibition-mediated increase of firing. Similarly, if the connection P→A is too strong, the activity of *A* cells could increase on activation of *P* cells (for a complete list of requirements, see Materials and Methods). In Constructing the spiking network, we explain how these requirements can be taken into account to constrain the spiking model.

**Figure 10.**
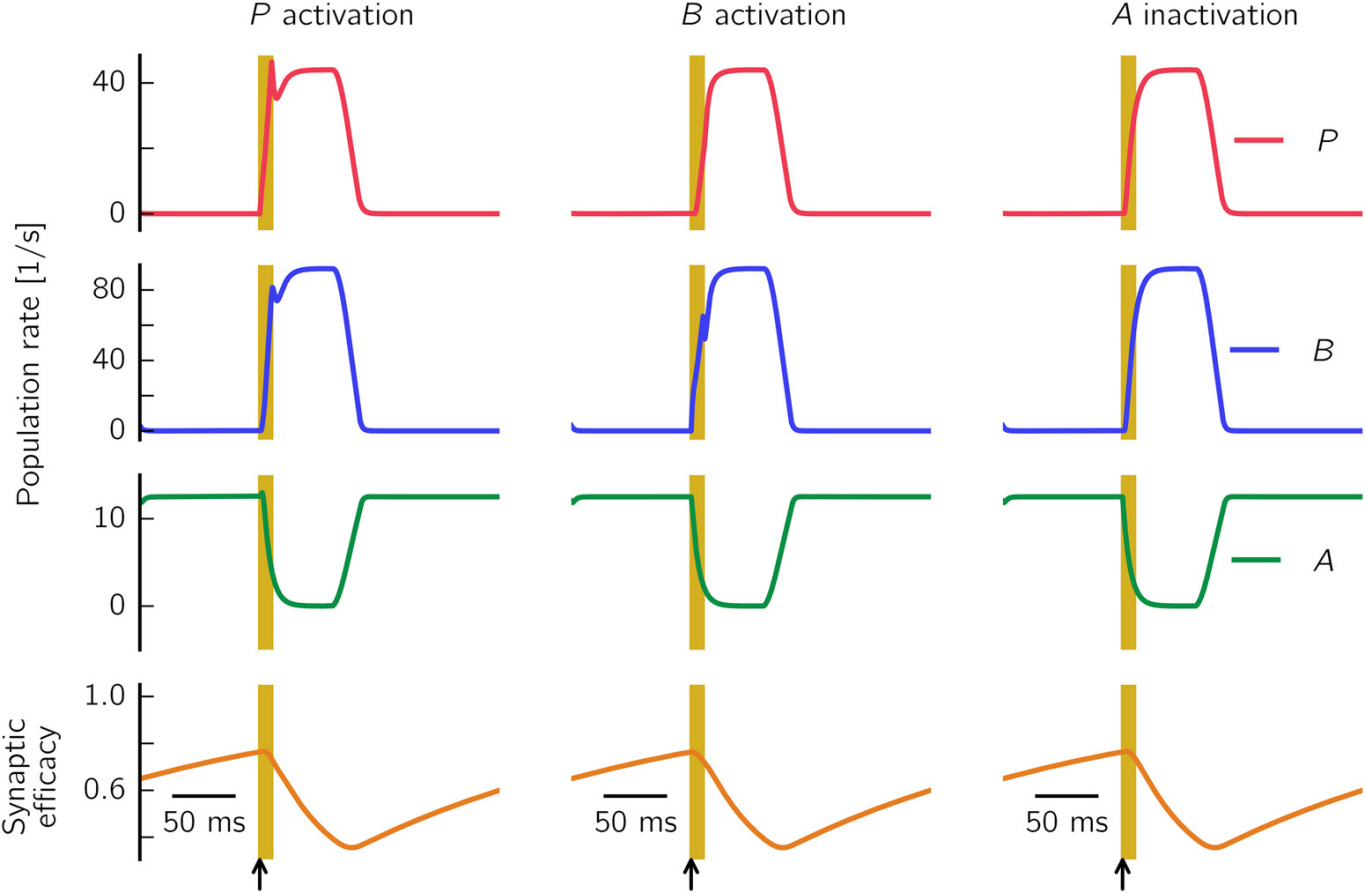
Rate network simulations with fitted softplus f-I curves display SWR-like events. Top to bottom, *P*, *B*, and *A* firing rates, and level of synaptic efficacy. Plot represents the time-dependent behavior on current injection when the synaptic efficacy dynamics is not clamped, but can evolve as stated in Equation 5. In each column, current is injected to a different population (left to right, *P*, *B*, and *A*). Currents are injected for a duration of 10 ms and are chosen to minimize the transient effects, yielding *I_P_* = 60 pA, *I_B_* = 150 pA, and *I_A_* = 200 pA. In all cases, switching on the current (yellow regions and arrows) makes the system switch to the SWR state (transient increase of *P* and *B* activity, and transient decrease of *A* activity). The depression mechanism (bottom) brings the system from a SWR state back to the non-SWR state. Results are comparable with the population firing rates of the spiking network presented in Figure 9*B–D*. Network parameters are summarized in Table 5.

As already motivated at the end of the previous section of Results, to better understand the dynamics of SWRs, we approximated the spiking model to a rate model (see also the Rate model). First, Figure 10 shows that current stimulation (to *P*, *B*, and *A* cells, from left to right) induces SWR events comparable to the results in the spiking model. Additionally, the rate-model approximation of the spiking model allows for a deeper understanding of these requirements and their impact on bistability. In Pathway strengths and quantification of requirements, we show that the rate-model approximation of the spiking model reveals its underlying dynamics (Fig. 6). Furthermore, we derived an explicit formulation of the conditions on the average connection strengths among the three populations of neurons. As a result, we were able to identify the dependence of bistability on the values of single connection strengths (Fig. 7). We could also show parameter combinations for which the network is both bistable, and with the expected behavior on current stimulation (increase of *P* and *B* firing, decrease of *A* firing); in Figure 8, these regions are shown as a function of pairs of parameters (green hatched regions). The comparison with two-parameter bifurcation diagrams (dark gray regions) confirms the existence of bistable networks with the desired effect of stimulation for multiple parameter combinations. Another main contribution of the bifurcation analysis displayed in Figures 6–8 is to demonstrate that the desired network behavior does not require parameter fine-tuning.

### Features of spontaneous and evoked SWRs match experimental results

A puzzling feature of spontaneously occurring SWRs is the coexistence of two different timescales: while a single event lasts 50-100 ms, the incidence of events is in the range of 1-2/s (depending on preparation and recording site, Buzsáki, 2015). Interestingly, Kohus et al. (2016) reported a strong correlation between the amplitude of SWR events and the interval to the previous SWR (IEI), recorded in the LFP of the CA3, *in vitro* (for similar results in CA1, see Jiang et al., 2018).

**Figure 11.**
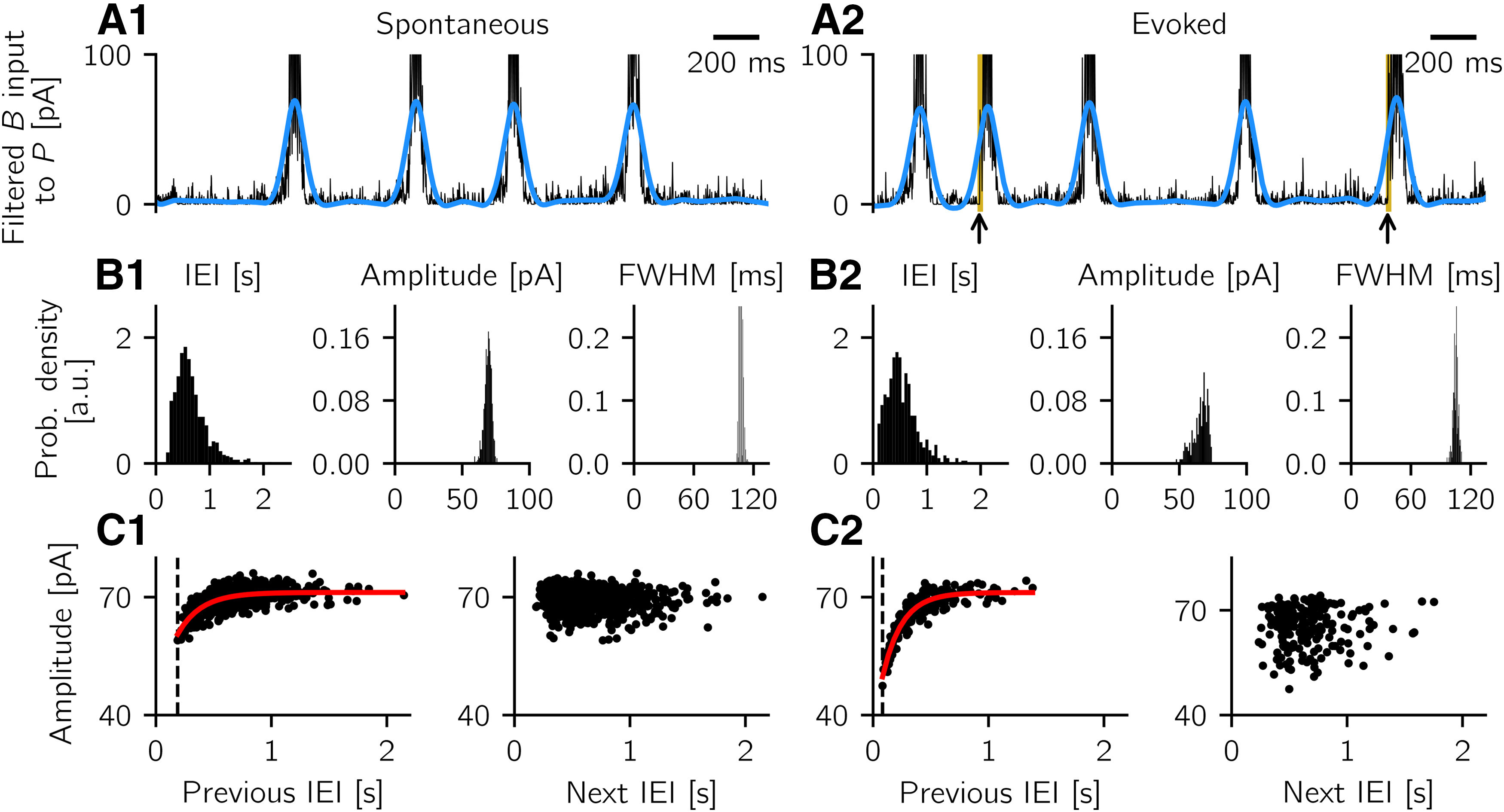
Properties of spontaneous and evoked SWRs. Left, Analysis of spontaneous events. Right, Analysis of evoked events. ***A1***, Mean *B* input current to *P* cells (sign-reversed, black trace) and its low-pass filtered (up to 5 Hz) version (blue trace). ***B1***, Properties of spontaneous SWRs: IEI (distance from end to start of events, where start and end points are calculated at half maximum of the filtered signal), amplitude of filtered events, and FWHM (see Materials and Methods). ***C1***, Left, Strong correlation between event amplitude and previous IEI. Each dot indicates a pair. Red line indicates the best fit exponential function (fitted time constant: 203 ms). Dashed line indicates the smallest observed IEI (188 ms). Right, Weak correlation between amplitude of event and length of the next IEI. ***A2***, ***B2***, ***C2***, Same as in ***A1***, ***B1***, and ***C1***, but for events evoked by current stimulation to *B* cells (as in Fig. 9*C*, current is injected for 10 ms, black arrows and yellow areas in ***A2***). ***C2***, Dashed line indicates the smallest observed IEI (82 ms), and the best fit exponential function has a time constant of 168 ms. Correlation results are in line with experimental observations (Kohus et al., 2016; Chenkov, 2017; Jiang et al., 2018). Parameters used to simulate the spiking network are listed in Tables 1–3.

To test whether the spiking network can reproduce these experimental results regarding the dynamics of SWR generation and termination (including the correlation structure across consecutive events), we simulate many SWRs (approximately 10 min of simulated time); in this simulation, SWR events occur spontaneously (incidence of ∼1.3/s). We use the low-pass filtered LFP signal (i.e., the average input current from *B* cells to *P* cells filtered up to 5 Hz, see Materials and Methods for details) to compare simulated SWRs to experimental SWRs. Examples of the raw and low-pass filtered traces of input current from *B* to *P* cells during spontaneous and evoked SWRs are shown in the upper row of Figure 11. Properties of spontaneous SWRs in the network are shown in Figure 11*B1*. The IEI distribution (mean: 0.65 s, SD: 0.28 s) is close to experimental results (Schlingloff et al., 2014; Jiang et al., 2018), in which the IEIs are in the range of 0.5-1 s. The amplitude of the SW event (69.15 ± 0.28 pA) and the FWHM (107.20 ± 1.73 ms) cannot be easily compared with experiments because our measure of the LFP is only an approximation of the recorded extracellular signal (see Materials and Methods). However, both SW amplitude and duration are quite variable over the course of a recording (Davidson et al., 2009; Ellender et al., 2010; Sullivan et al., 2011; Hofer et al., 2015; Levenstein et al., 2019; Fernández-Ruiz et al., 2019), similar to the results shown in Figure 11*B1*. Furthermore and in agreement with others (Kohus et al., 2016; Chenkov, 2017; Jiang et al., 2018), we observe a strong correlation (Pearson correlation coefficient *c* = 0.57, $p=1.71\cdot 10^{-68}$) between the event amplitude and the length of the previous IEI, as shown in Figure 11*C1* (compare with Kohus et al., 2016, their Fig. 13*A*; as no correlation coefficients are given, we rely on visual inspection). Interestingly, the correlation between the event amplitude and the length of the next IEI is low (Pearson correlation coefficient c=−0.06,p=0.079). The lack of correlation between event amplitude and next IEI has been reported by Chenkov (2017, his Fig. 3.5) in experimental data obtained in CA3 *in vitro*. Finally, we also observe a correlation between the duration of a SWR event and the length of the previous (but not next) IEI (Pearson correlation coefficient *c* = 0.168, $p=2.18\cdot 10^{-6}$), as predicted by theoretical results on noise-induced transitions to excitable states (Lim and Rinzel, 2010). To the best of our knowledge, however, the existence of this correlation has not been described in experiments.

Optogenetic drive can elicit SWRs with shorter IEIs than the spontaneous events, but with a similar correlation structure between IEI and amplitude (Kohus et al., 2016). To simulate such experiments, we additionally consider the case of evoked events. The right column of Figure 11 shows that the spiking network reproduces the experimentally observed behavior. In these simulations, SWRs occur spontaneously but are additionally triggered by stimulation of *B* cells (similar to Kohus et al., 2016, their Fig. 13*C*). A short snapshot of the simulation is shown in Figure 11*A2*. Figure 11*B2* shows the properties of evoked events. Evoked SWRs are all-or-none events, with IEI distribution, amplitude, and FWHM similar but slightly more variable compared with spontaneous events (compare with Fig. 11*B1*). Figure 11*C2* shows the presence of a strong correlation between the amplitude of evoked SWRs and the length of the previous IEI (Pearson correlation coefficient *c* = 0.77, $p=7.75\cdot 10^{-43}$), but not with the next IEI (Pearson correlation coefficient *c* = 0.01, *p* = 0.889). Only the amplitude of evoked events and the interval to the previous or next SWRs are used in this analysis.

The simulations shown in Figure 11 also reveal the existence of a refractory period following a spontaneous SWR event (dashed line at ∼188 ms in Fig. 11*C1* indicates the smallest observed IEI). A refractory period is in line with results obtained by others (Schlingloff et al., 2014; Kohus et al., 2016; Jiang et al., 2018). The duration of the refractory period is expected to correlate with the strength of the stimulation, which also explains why evoked events can be triggered already at ∼82 ms following the previous event (Fig. 11*C2*, dashed line).

**Figure 12.**
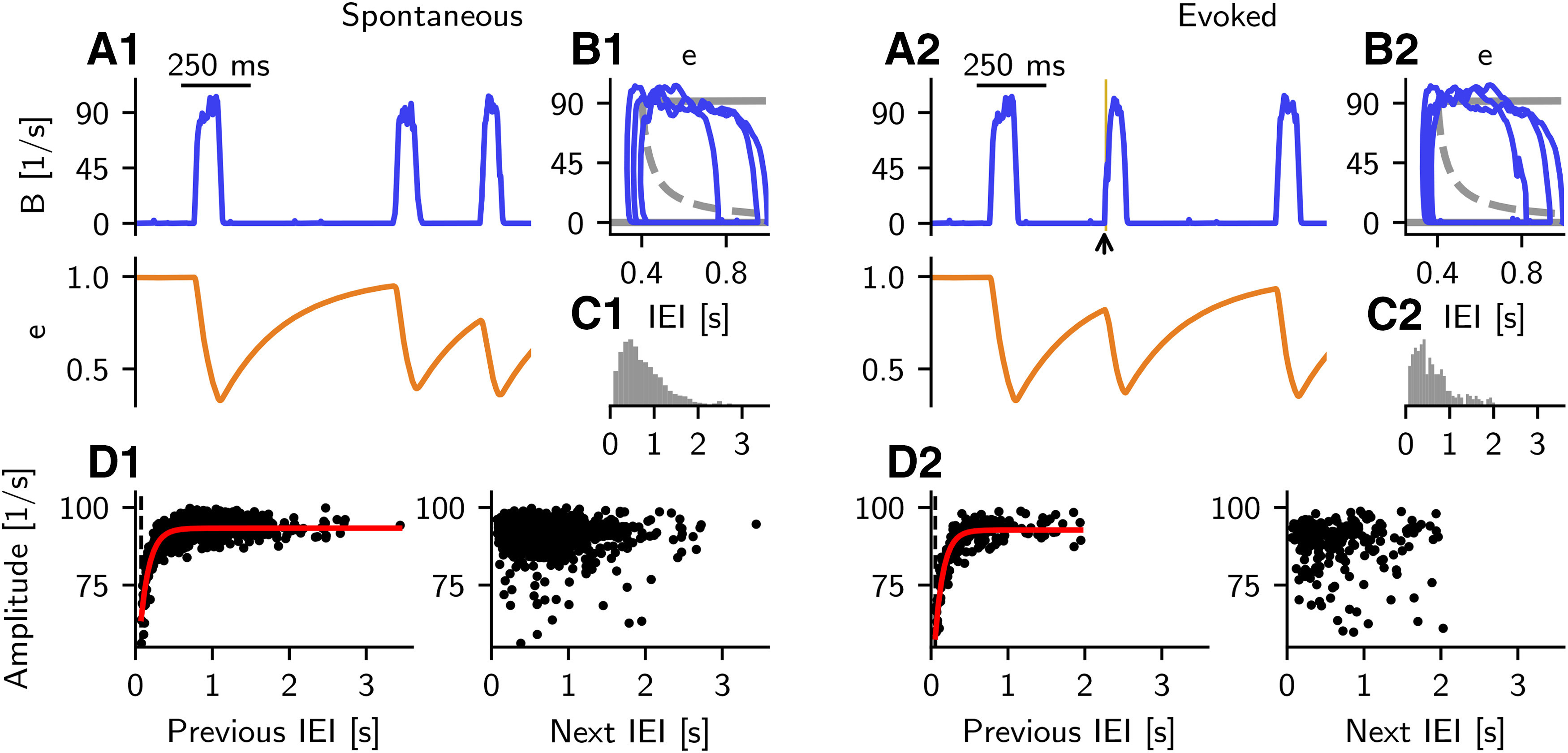
Properties of spontaneous and evoked events in the rate model with noise. Noisy inputs mimicking synaptic current updates are injected to each rate-model population (see Materials and Methods). Left, Analysis of spontaneous events. Right, Analysis of evoked events. ***A1***, *B* population rate (blue trace) and synaptic depression *e* (orange trace) displayed for 1.5 s of simulation. ***B1***, *B-e* phase-plane view of trajectories shown in ***A1***, overlain by the corresponding bifurcation diagram (gray). ***C1***, Histogram of IEI (distance from end to start of events, calculated at half maximum) of spontaneous events. Events are calculated from the low-pass filtered (up to 10 Hz) *B* trace. ***D1***, Amplitude (calculated from filtered signal) of each event with respect to the IEI. Left, Strong correlation between event amplitude and previous IEI. Each dot indicates a pair. Red line indicates the best fit exponential function (fitted time constant: 119 ms). Dashed line indicates the smallest observed IEI (74 ms). Right, Weak correlation between amplitude of event and length of the next IEI. ***A2***, ***B2***, ***C2***, ***D2***, Same as in ***A1***, ***B1***, ***C1***, and ***D1***, but for events evoked by additional step-current stimulation to *B* population (as in Fig. 10, currents are injected for a duration of 10 ms with amplitude *I_B_* = 150 pA; as in Fig. 11, currents are injected at intervals of ∼2 s; black arrow and yellow line in ***A2***). ***D2***, Dashed line indicates the smallest observed IEI (55 ms), and the best fit exponential function has a time constant of 110 ms.

The results presented above for the spiking network can be replicated in the rate-model approximation (Fig. 12). In these simulations, noisy inputs have been added to the (otherwise deterministic) rate model; this noise mimics the variability of the inputs to each population in the non-SWR state (see Rate-model noise). Figure 12*A1* presents a short segment of the time course of the *B* population activity and of the synaptic efficacy variable *e* (out of a 10 min simulation). The noisy inputs are sufficient to trigger spontaneous SWRs with an incidence of ∼1.2/s. Figure 12*B1* shows the same simulation in the *e*-*B* phase plane (similar to the central plot in Fig. 6*D*), where we can observe that the unstable fixed point (dashed branch) can be overcome at different values of *e*. Figure 12*C1* indicates that the IEI distribution of the rate model is comparable to that of the spiking model (mean = 0.76 s, SD = 0.51 s; compare with Fig. 11*B1*). Finally, Figure 12*D1* shows that the correlation structure of SWR events with previous and next IEI is preserved: the left plot reveals the existence of a refractory period and a correlation between SWR amplitude and the length of the previous IEI (Pearson correlation coefficient *c* = 0.47, $p=7.36\cdot 10^{-41}$; compare with Fig. 11*C1*), whereas the right plot demonstrates that the SWR amplitude does not correlate with the length of the next IEI (Pearson correlation coefficient *c* = –0.05, *p* = 0.224). As in the spiking network simulations, the duration of the events (given by the FWHM of the filtered signal) is also correlated with the length of the previous (but not next) IEI (Pearson correlation coefficient *c* = 0.58, $p=1.78\cdot 10^{-64}$; data not shown). In addition, we study the structure of evoked SWRs in the rate model: Figure 12*A2*, *B2* depicts a short segment of simulation in which SWRs can either arise spontaneously (because of noisy inputs) or be elicited by current injection to *B* cells (see Quantification of SWR properties in the noisy rate model). Figure 12*C2* shows that the IEI distribution of evoked SWRs is comparable to that of spontaneous events in the rate model (mean = 0.62 s, SD = 0.44 s). Finally, Figure 12*D2* confirms the existence of a strong correlation between the amplitude of evoked SWRs and the length of the previous IEI (Pearson correlation coefficient *c* = 0.57, $p=1.93\cdot 10^{-20}$), but not with the length of the next IEI (Pearson correlation coefficient *c* = –0.04, *p* = 0.524). To summarize, simulations of the rate model with noise can replicate the main features of simulations of the spiking model, such as the high similarity of spontaneous and evoked SWRs, their refractoriness, and a characteristic correlation between IEI and event amplitude.

**Figure 13.**
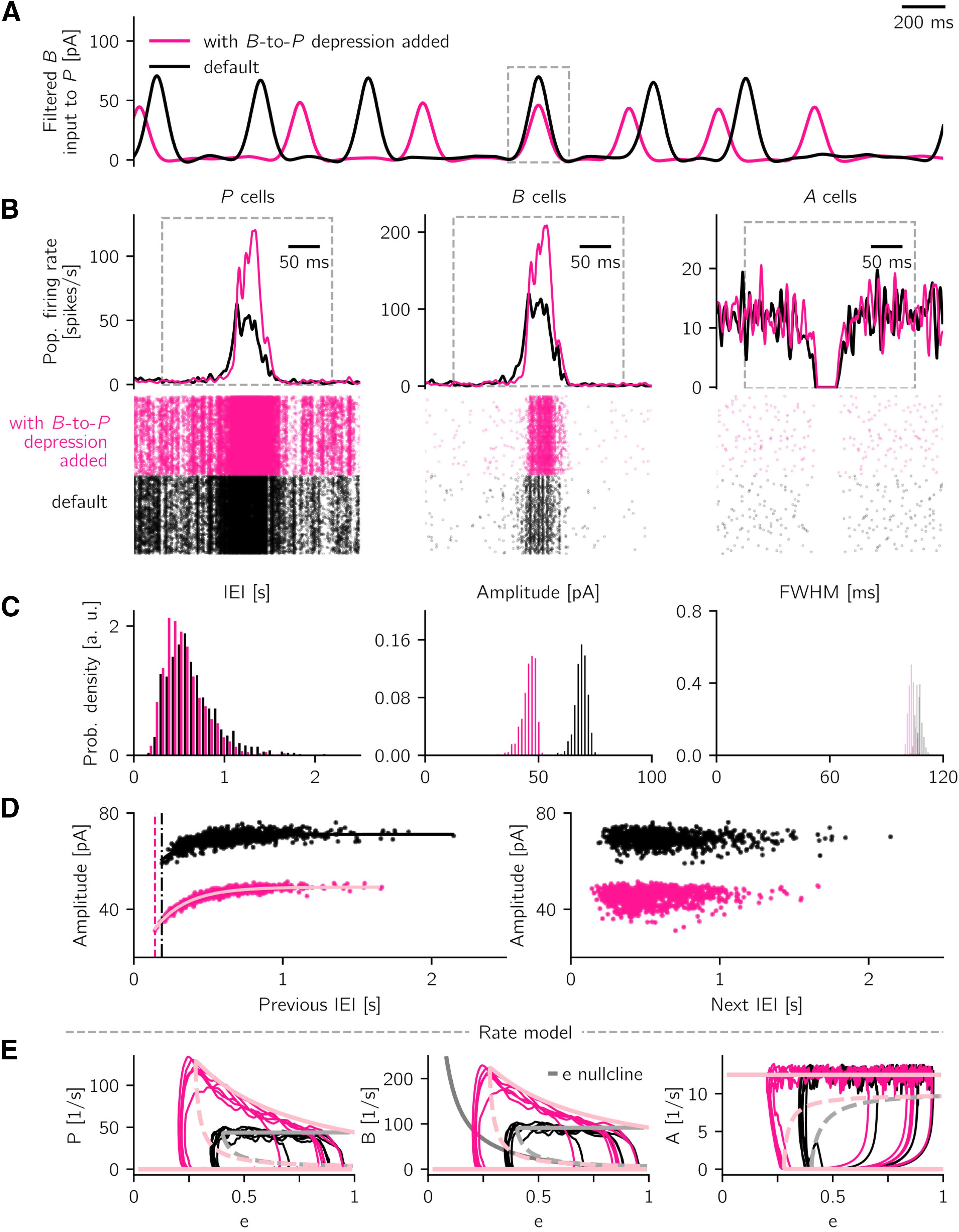
Effect of additional PV^+^ BC-to-pyramidal cell synaptic depression. ***A***, Snapshot of spontaneous, low-pass (<5 Hz) filtered LFP activity in default setting (black, as Fig. 11) and in the scenario where B→P synaptic depression is added (pink). ***B***, One event is isolated, and the corresponding population firing rates and cells' raster plots are shown (for *P*, *B*, and *A* cells, respectively). Events are aligned with respect to the peak of the LFP signal. ***C***, Properties of spontaneous SWR events are summarized in histograms (IEI; amplitude; FWHM), in default (black) and B→P depression (pink) scenarios. ***D***, Correlation structure of sharp wave amplitude and previous (left) and next (right) IEI are remarkably similar in the two scenarios. The shift along the vertical axis is caused by the decreased event amplitude in the case with B→P depression. Dashed lines indicate the smallest observed IEI for the default case (188 ms, black) and the case with additional B→P depression (142 ms, pink). Solid curves indicate best fit exponential functions (fitted time constants are τ = 203 ms in default case and τ = 214 ms in the case with added *B*-to-*P* depression). Parameters used to simulate the spiking network are listed in Tables 1–3 and in Short-term plasticity. ***E***, Rate-model bifurcation diagrams show the steady-state rates of *P*, *B*, and *A* as a function of the synaptic efficacy *e* in default scenario (light gray) and with additional B→P depression (light pink). Solid (dashed) light pink and light gray curves indicate stable (unstable) fixed points. Middle, Solid dark gray curve indicates the *e*-nullcline, given by the last line in Equation 5. This synaptic depression mechanism causes *e* to increase in the non-SWR state, which allows fluctuations to start a SWR event, and causes *e* to decrease in the SWR state, which terminates the SWR event. Overlain are traces of a 3 s simulation of the rate model with noise (see Rate-model noise) for default case (black) and with additional B→P depression (pink). During a SWR, the additional B→P depression leads to increasing *P* and *B* activity while *e* is decreasing (curved shape of the SWR state in the left and middle panels). Network parameters are summarized in Table 5.

In the context of our models, the correlation structure between IEIs and amplitudes of SWRs can be explained by the dynamics of the synaptic depression at the B→A connection. During a SWR, high activity of *B* cells decreases the efficacy of the B→A connection (see Eq. 3). Whenever the synaptic efficacy decreases to a critical minimal value ($e^{AB}=0.38\pm 0.01$, range [0.35, 0.40] in the spiking simulations), the inhibition at B→A synapses becomes ineffective, and this induces the termination of a SWR event by restoring the high activity of *A* cells. The existence of a critical value of synaptic efficacy below which the SWR state disappears is confirmed by the bifurcation analysis displayed in Figure 6*D* (see also Fig. 12*B1*, *B2*). As active *A* cells successfully inhibit *B* cells in the non-SWR state, the synaptic efficacy recovers; once it is well above the critical value, fluctuations in *B* cell activity can trigger a new SWR. The value of the synaptic efficacy at the beginning of a SWR ($e^{AB}=0.85\pm 0.04$, range [0.70, 0.93] in the spiking simulations) depends on the length of the recovery time, which in turn controls the number of *B* spikes needed to reach the critical value during a new SWR. Thus, longer recovery times mean that the synaptic efficacy at the beginning of the SWR is large, and more *B* spikes are needed to reach the critical termination value. As a result, we expect the amplitude of a SWR event (mean *B* input current to P cells, see Materials and Methods) to correlate with the length of the previous IEI. Conversely, the time to the next event is determined by the recovery of the synaptic efficacy variables, which starts from a value that exhibits low variability. Thus, the amplitude of an event should not influence the interval to the next spontaneous SWR, suggesting a low correlation between the event amplitude and the length of the next IEI. Finally, the recovery from depression also explains the existence of a refractory period during which no SWRs are generated: shortly after a SWR, the synaptic efficacy of B→A connections is too weak for *B* cell activity to suppress *A* cells and trigger a new SWR.

### Additional short-term plasticity mechanisms

Up to this point, we have used a spiking model that includes a minimal set of components, which were sufficient to reproduce the experimental findings of interest. We are thus undoubtedly neglecting many other phenomena, which might also contribute to the modulation of SWR dynamics. For example, it is well known that, both in the hippocampus and neocortex, synapses from PV^+^ BCs to pyramidal cells are depressing (Galarreta and Hestrin, 1998; Kraushaar and Jonas, 2000; Szabó et al., 2010; Kohus et al., 2016). Another prominent plasticity mechanism is the short-term facilitation at synapses connecting pyramidal cells to different types of interneurons (Reyes et al., 1998; Wierenga and Wadman, 2003; Silberberg and Markram, 2007; Pala and Petersen, 2015; English et al., 2017; Nanou et al., 2018). In the hippocampus, this mechanism has been mostly investigated for oriens-lacunosum-moleculare cells (Ali and Thomson, 1998; Losonczy et al., 2002; Böhm et al., 2015). Although the identity of anti-SWR cells is currently unknown, this property could be nevertheless interesting to consider in the network. To get a better intuition of the impact of short-term plasticity on SWR dynamics, we thus investigate the effect of B→P depression and P→A facilitation in the spiking network. The results presented in Bifurcation analysis of rate model indicate that the other connections are not well suited for a dynamic control of SWRs in our model.

#### B→P synaptic depression

First, we test the effect of an additional short-term B→P synaptic depression in the model, and compare the results with the default case (i.e., with the case in which the B→A synapses are the only plastic connections). For simplicity, we assume that the properties of B→P depression (time decay and plasticity rate) are identical to those of the B→A depression. This assumption is motivated by the fact that both mechanisms share the same presynaptic population (for details about how to model synaptic depression, see Eq. 3).

As a result of B→P synaptic depression, *B* inhibition onto pyramidal cells is reduced. How does this impact SWRs in our setup? As in the default scenario with plastic B→A connection, the depression gets markedly activated during a SWR event, when *B* cells increase their firing rate. Hence, *P* cells receive less inhibition while being already active. This suggests, for example, that the population rate of *P* cells increases while the B→P depression is on. This behavior is confirmed in Figure 13, which shows simulations of the spontaneous network when both depression mechanisms are active. Figure 13*A* shows that the approximated sharp wave signal has lower amplitude when the B→P depression is present. Given that the LFP is defined as a low-pass filtered version of the mean *B* input to *P* cells (Materials and Methods), this effect is not surprising. The reduced *B* input to *P* cells also results in an increase of the population firing rate of *P* cells (Fig. 13*B*, left). Because of this increased activity of *P*, the activity of *B* is also increased (Fig. 13*B*, middle), but this increase does not balance (in the LFP) the depression of B→P. The activity of *A* cells remains very low during the SWR, and is basically unchanged outside of the SWR (Fig. 13*B*, right). The bifurcation analysis of the rate model shown in Figure 13*E* corroborates the effects of the additional B→P depression on the population firing rates: in the SWR state, decreasing *e* increases *P* and *B* (pink upper branches in left and middle plots) but does not change *A* (pink lower branch in right plot). In the default scenario (Fig. 13*E*, black traces), population rates in the SWR state are independent of the exact value of *e* (inside the bistable region).

The properties of the approximated sharp wave signal are quantified in Figure 13*C*. As discussed, B→P depression decreases event amplitudes, and the increased *B* activity does not compensate for this. Interestingly, the IEIs remain largely unaffected. The FWHM is slightly lower in the scenario with B→P depression. However, it is important to keep in mind that the FWHM is intrinsically linked to the event amplitude; thus, it can be misleading to compare it across conditions where events have different amplitudes. Finally, Figure 13*D* shows that the correlation structure of SW amplitude and previous or next IEI stays remarkably unchanged when the B→P depression is added (Pearson correlation coefficient for case with B→P depression: amplitude and previous IEI: *c* = 0.81, $p=2.85\cdot 10^{-204}$, amplitude and next IEI: *c* = 0.04, *p* = 0.204). Overall, we conclude that the network properties are largely preserved when a B→P depression mechanism is added to the default network with B→A depression.

Could the B→P depression replace the B→A depression in the network? In a network with B→P depression alone (i.e., nonplastic B→A connections), *P* cells receive less inhibition when they are active (during a SWR), and thus persist in an active state. Hence, the network cannot escape from the SWR state, and events do not terminate. The bifurcation analysis of the rate model can explain this (Fig. 7, B→P): a decrease in *W_PB_* alone cannot bring the system away from bistability). In this sense, the B→P depression can be thought of as an additional, but not alternative, mechanism to the B→A depression.

#### P→A synaptic facilitation

To test the effect of P→A facilitation, we compare the behavior of the default network with the one of a network to which this mechanism is added (for details about the implementation, see Materials and Methods). Short-term facilitation at P→A synapses is expected to increase the excitation seen by the *A* cells when *P* cells are active (i.e., during a SWR). Thus, this mechanism supports the termination of SWRs by restoring the high firing rate of *A* cells.

**Figure 14.**
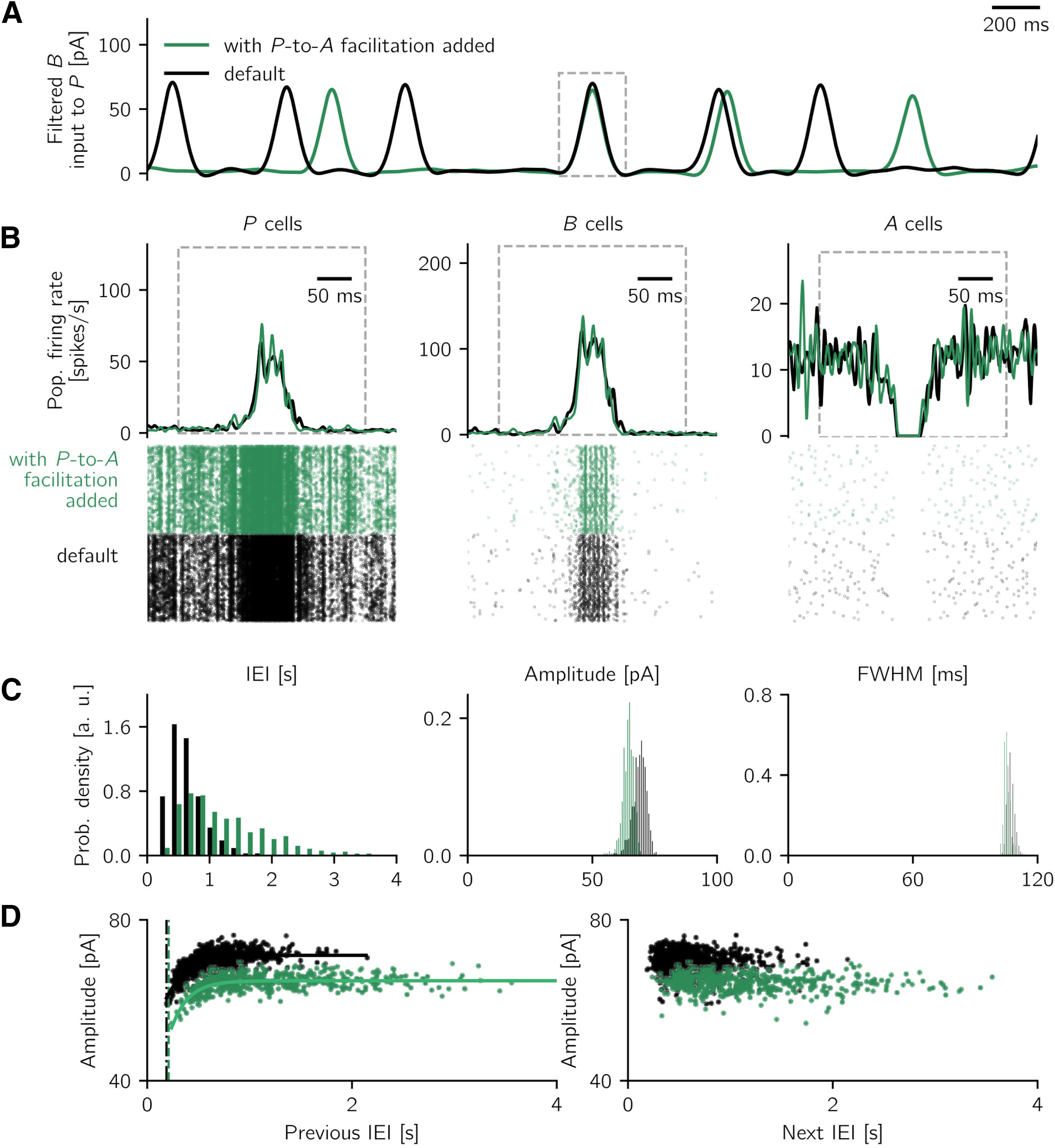
Effect of additional pyramidal-to-anti-SWR cell synaptic facilitation. ***A***, Snapshot of spontaneous, low-pass filtered (<5 Hz) LFP activity in default setting (black, as Fig. 11) and in the scenario where P→A synaptic facilitation is added (green). ***B***, One event is isolated, and the corresponding population firing rates and cells' raster plots are shown (for *P*, *B*, and *A* cells, respectively). Events are aligned with respect to the peak of the LFP signal. ***C***, Properties of spontaneous SWR events are summarized in histograms (IEI; amplitude; FWHM), in default (black) and P→A facilitation (green) scenarios. ***D***, Correlation structure of sharp wave amplitude and previous (left) and next (right) IEI are remarkably similar in the two scenarios. The shift along the vertical axis is caused by the decreased event amplitude in the case with P→A facilitation. Dashed lines indicate the smallest observed IEI for the default case (188 ms, black) and the case with additional P→A facilitation (209 ms, green). Solid curves indicate best fit exponential functions (fitted time constants are τ = 203 ms in default case and τ = 214 ms in the case with added *P*-to-*A* facilitation). Parameters used to simulate the spiking network are listed in Tables 1–3 and in Short-term plasticity.

The facilitation effects in the network are summarized in Figure 14. The amplitude of the LFP signal is slightly reduced (Fig. 14*A*,*C*), an effect that is related to a stronger A→B inhibition caused by slightly more active *A* cells. Figure 14*B* shows that the population firing rates of *P* and *B* cells are virtually unchanged in the case with P→A facilitation, in line with the bifurcation analysis of the rate model (Fig. 7). Additionally, the IEI distribution is slightly shifted to larger values in the case of added P→A facilitation because the recovery of both B→A depression and P→A facilitation is needed to start a SWR event. However, Figure 14*D* (dashed lines) shows that the refractoriness is largely controlled by the B→A depression. Interestingly, the correlation structure in Figure 14*D* shows a similar trend as the default scenario (Pearson correlation coefficient for case with facilitation: amplitude and previous IEI: *c* = 0.48, $p=3.97\cdot 10^{-37}$, amplitude and next IEI: *c* = –0.02, *p* = 0.567). Overall, we can conclude that the network is robust to the addition of a P→A facilitation mechanism.

**Figure 15.**
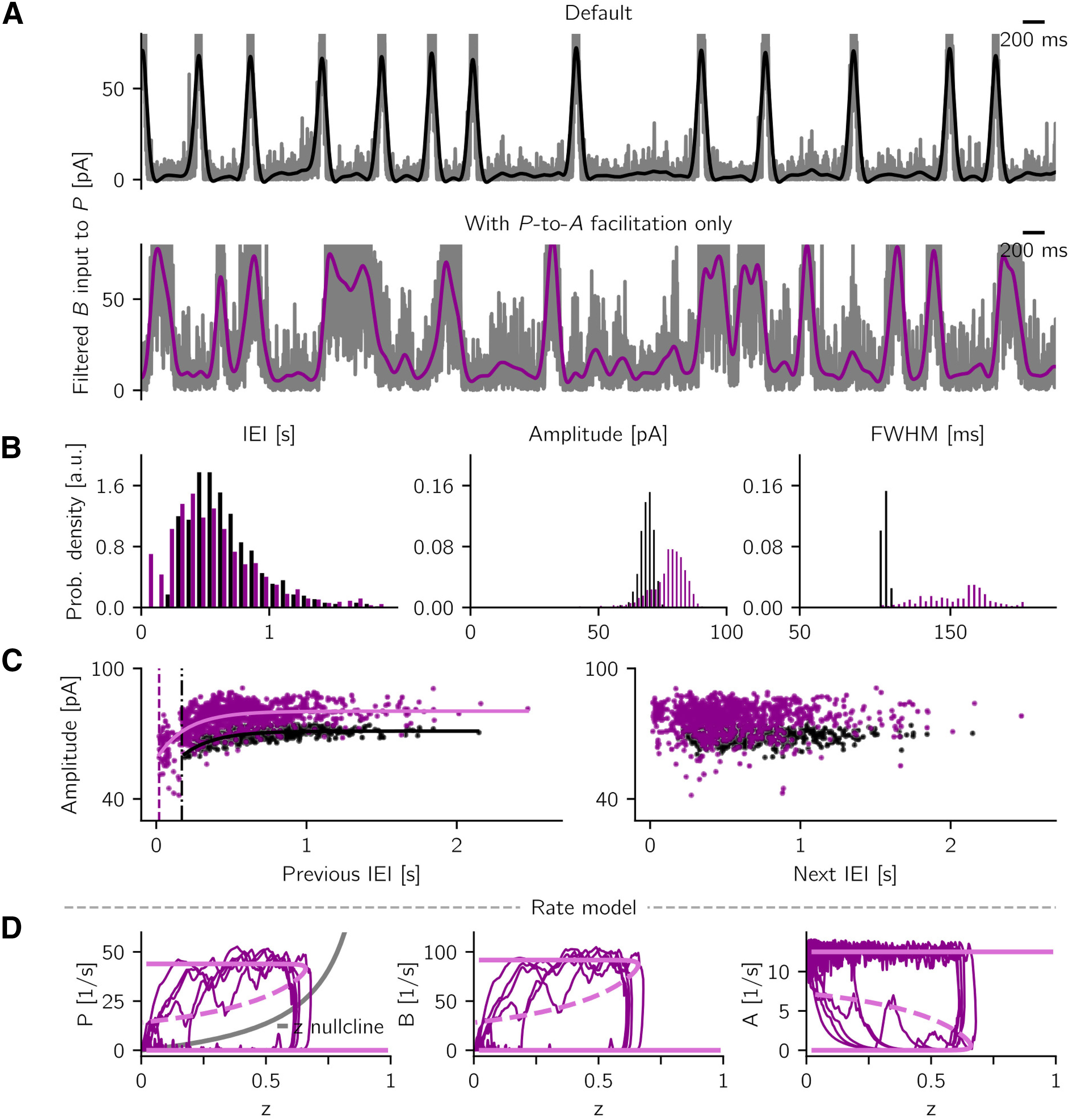
Pyramidal-to-anti-SWR cells synaptic facilitation can regulate SWR initiation and termination in the network. ***A***, Snapshot of spontaneous, low-pass filtered (<5 Hz) LFP activity in default setting (black, as Fig. 11) and in the scenario with P→A synaptic facilitation alone (i.e., no B→A depression, purple). ***B***, Properties of spontaneous SWR events are summarized in histograms (IEI; amplitude; FWHM), in default (black) and facilitation-only (purple) scenarios. Note the wider distribution of FWHM and the short IEIs (<100 ms) in the facilitation-only scenario. ***C***, Correlation structure of sharp wave amplitude and previous (left) and next (right) IEI is preserved in the scenario with P→A facilitation alone. Dashed lines indicate the smallest observed IEI for the default case (188 ms, black) and the case with P→A facilitation (19 ms, purple). Solid curves indicate best fit exponential functions (fitted time constants are τ = 203 ms in default case and τ = 200 ms in the *P*-to-*A* facilitation-only case). In the simulation with P→A facilitation, the reciprocal connections among interneurons are adjusted to yield enough events (see Short-term plasticity). All other parameters are listed in Tables 1–3. ***D***, Rate-model bifurcation diagrams represent the steady-state rates of *P*, *B*, and *A* as a function of the efficacy *z* of the connection P→A (see Short-term plasticity in the rate model). Solid and dashed light pink curves indicate stable and unstable fixed points, respectively. The system is bistable for *z* < 0.66. Left, Solid gray curve indicates the *z*-nullcline. This synaptic facilitation mechanism causes *z* to decrease in the non-SWR state, which enables fluctuations to start a SWR event, and to increase in the SWR state, which terminates the SWR event. Overlain are traces of 8 s simulation of rate model with noise (see Rate-model noise) with P→A facilitation only. For these calculations, we fixed *e* = 0.5 in the B→A connection. Further parameters are summarized in Table 5.

Could the P→A facilitation replace the B→A depression in the network? To investigate this case, we simulate a network where the P→A facilitation is the only plastic mechanism in the network (i.e., the synaptic efficacy of the B→A connection is clamped at *e^AB^* = 0.5 for the whole duration of the simulation). Figure 15*A* shows that spontaneous events emerge in such a network. Events have a much longer duration and larger variability (as indicated by the FWHM) than the ones in the default network (Fig. 15*B*, right); however, events can occur with much shorter IEI than the default case (Fig. 15*B*, left, purple bars with IEI < 100 ms). This can be explained by recognizing that, in the network with facilitation only, the initiation and termination mechanisms are distinct. An event is initiated when fluctuations at *B* cells are large enough to inhibit the activity of *A* cells. For this, the B→A connection needs to be strong (little or no depression). After an event has started, the facilitation increases the efficacy of the P→A connection, which can move the network out of the bistable regimen and thus terminate the SWR. Meanwhile, fluctuations in *B* can still prompt the inhibition of *A* cells. Thus, the *A* cells get a mixed signal (inhibition from *B* and excitation from *P*), which can prolong the time required for the facilitation to make the *A* cells fully active again, and thus to terminate a SWR event. After a SWR is terminated, a new SWR could be initiated with virtually no refractoriness because the rate (and fluctuations) in *B* are strong straightaway. Conversely, in the default scenario (B→A depression only), the initiation and termination mechanisms are both dependent on the B→A connection, leading to a lower variability of FWHM by preventing the occurrence of longer events and giving rise to stronger refractoriness. In other words, in the default case, the effect of possible fluctuations in the activity of *B* cells during and immediately after SWRs is suppressed by the (depression-driven) lower efficacy of the B→A connection, and new events cannot be triggered before the depression has recovered.

A remarkable feature of the simulations with only P→A facilitation is that the network can still reproduce a strong correlation between event amplitude and previous IEI as experimentally observed by Kohus et al. (2016) (Fig. 15*C*; Pearson correlation coefficient, amplitude and previous IEI: *c* = 0.41, $p=7.45\cdot 10^{-38}$, amplitude and next IEI: *c* = 0.08, *p* = 0.020). This result suggests that the SWR termination mechanism is a main component influencing the existence of the correlation between IEIs and SWR amplitude. Finally, the analysis of a rate-model approximation of the spiking model (with fixed *e* = 0.5 and dynamic P→A facilitation) confirms the existence of bistability in this scenario. Figure 15*D* shows that for a wide range of values of the facilitation variable *z*, non-SWR and SWR states coexist, which is similar to the default scenario with B→A depression (compare these plots with the black traces in Fig. 13*E*).

To conclude, we have shown in this section how additional short-term plasticity mechanisms affect the dynamics of SWRs. We have focused on the depression B→P and on the facilitation P→A, which were shown to preserve the main features of SWRs. Moreover, we have shown that P→A facilitation can replace the default depression at the B→A connection in generating spontaneous SWRs with the right correlation structure. However, in the P→A facilitation-only scenario, SWRs lack the refractory period typically observed in experiments (Schlingloff et al., 2014; Kohus et al., 2016; Jiang et al., 2018; Levenstein et al., 2019). This suggests that the P→A facilitation alone is not sufficient to reproduce SWR-like activity.

Overall, if *A* cells are mediating disinhibition in CA3, our results predict that the B→A depression is a key mechanism controlling the initiation and termination of SWRs.

## Discussion

We have shown that a spiking network consisting of pyramidal cells and two types of interneurons (PV^+^ BCs and a class of anti-SWR cells), equipped with a short-term synaptic depression at the synapses connecting PV^+^ BCs to anti-SWR cells, is able to generate SWRs and to reproduce multiple features of experimentally recorded SWRs. SWRs can emerge spontaneously in the network or can be triggered by cell stimulation (activation of pyramidal or PV^+^ BCs, or inactivation of anti-SWR cells). The crucial mechanism underlying this behavior is the disinhibition of pyramidal cells via suppression of anti-SWR cells by active PV^+^ BCs. The model thus predicts strong connections in the disinhibitory pathway from PV^+^ BCs to pyramidal cells via anti-SWR cells.

The model explains the paradoxical finding that PV^+^ cell stimulation (Schlingloff et al., 2014; Kohus et al., 2016) can trigger SWRs. In these studies, optogenetic activation acted on all PV-expressing cell types. In the model, we have assumed that a selective activation of PV^+^ BCs is sufficient to initiate a SWR event. The recruitment of PV^+^ BCs for SWR generation in the model is in line with experiments showing an involvement of inhibitory neurons during the initial phase of a SWR (Ellender et al., 2010; Sasaki et al., 2014; Bazelot et al., 2016). The model also reproduces the dynamics of spontaneous SWRs (Kohus et al., 2016; Chenkov, 2017; Jiang et al., 2018), in particular the existence of a strong correlation between SW amplitude and length of the previous (but not the next) IEI.

We predict the existence of a population of interneurons, the anti-SWR cells, which are tonically active in non-SWR states and stop firing during SWR events. We also predict that inactivating these cells is sufficient to trigger a SWR event. Which cell types could possibly represent anti-SWR cells? There are several possible candidates: interneurons recorded *in vivo* in the alveus and stratum oriens of CA1 decreased their firing during SWRs (anti-SPW cells in Csicsvari et al., 1999b). Fuentealba et al. (2008) reported the existence of an enkephalin-expressing GABAergic cell in CA1, *in vivo*, which seemed to be antimodulated with SWRs. Additionally, Le Van Quyen et al. (2008) showed that a subset of putative interneurons recorded in the human hippocampal formation stopped firing during the initial phase of a SWR event. Finally, Viney et al. (2013) identified CA3 axo-axonic cells that reduced their firing during SWRs (but see also Klausberger et al., 2003; Varga et al., 2012, 2014; Hájos et al., 2013). Despite these results, the identity of interneurons with antimodulated discharge properties is still unclear.

We propose that the connection from PV^+^ BCs to anti-SWR cells (B→A synapses) plays an important role in regulating the incidence of SWRs. PV^+^ BCs contact different classes of inhibitory neurons (Sik et al., 1995; Cobb et al., 1997; Kohus et al., 2016; Walker et al., 2016), but an experimental test of the existence and properties of the B→A connection relies on the identification of *A* cells. The choice of short-term synaptic depression at B→A synapses was inspired by Kohus et al. (2016), in which it was shown that SWR occurrence correlates with a depression mechanism from PV^+^ BCs to pyramidal cells.

Various other adaptation mechanisms could control the dynamics of SWRs. Figures 13–15 demonstrate robustness with respect to facilitation at P→A and depression at P→B, but these mechanisms cannot replace the depression at B→A. Aside from that, the bifurcation analysis (Fig. 7) indicates that short-term depression at P→B would be suitable; however, this synapse is governed by facilitation (Nanou et al., 2018). Alternatively, SWRs could be regulated by spike frequency adaptation (Kneisler and Dingledine, 1995; Povysheva et al., 2013; Ha and Cheong, 2017; Levenstein et al., 2019), for example, in the *P* or *B* cells, although it is currently unclear whether PV^+^ BCs express this property. Moreover, English et al. (2014) proposed that cellular hyperpolarization following a SWR event could induce a period where pyramidal cells are silent. This hyperpolarization could be the result of the activation of Ca^2+^-dependent or potassium currents (Zhang et al., 2006; Fano et al., 2012). Such additional adaptation mechanisms could help to prevent excessively long SWR-like activity, which could damage the biological network.

The model has been constructed such that, in the absence of dynamic short-term plasticity, SWR and non-SWR states coexist. Each state is dominated by an active pyramidal-interneuron subnetwork (see Fig. 2*B*). This bistable configuration relies on strong mutual connections between the two interneuron populations *A* and *B*, another critical model prediction. In a perfect bistable configuration, that is, when short-term plasticity is clamped at intermediate values (e.g., *e^AB^* = 0.5 in Fig. 2*A*), transitions between SWR and non-SWR states can be induced only by current injection but do not arise spontaneously. The addition of short-term depression at B→A synapses is sufficient to disrupt bistability: for large values of the synaptic efficacy, small fluctuations in the network activity can suffice to trigger a transient SWR event, which is terminated by the decrease of synaptic efficacy. This type of inhibitory networks, where noisy behavior and slow adaptive mechanisms coexist, has been studied previously (Moreno-Bote et al., 2007; Shpiro et al., 2007; Curtu et al., 2008; Shpiro et al., 2009; Jercog et al., 2017; Levenstein et al., 2019), but mostly in the scenario with only one or two populations. According to the terminology in Levenstein et al. (2019), our model corresponds to an excitableDOWN regimen; however, our model complements this work by providing a more mechanistic framework that can explain SWR generation and its dependence on interneuron activation (Schlingloff et al., 2014; Kohus et al., 2016).

In our approach, the network connectivity was set to have a bistable configuration. The bifurcation analysis presented in Materials and Methods shows that the studied networks are not the result of parameter fine-tuning, but rather representatives of a broad class of bistable disinhibitory networks. In biological networks, how could the connections be tuned so that all desired properties of the network are fulfilled? We propose that inhibitory spike timing-dependent plasticity (see Woodin et al., 2003; Vogels et al., 2011, 2013; Luz and Shamir, 2012) could be used to set the inhibitory-to-excitatory connections in each subnetwork. Future theoretical work could explore the feasibility of this approach.

The model presented in this study illustrates in great detail the complex interplay of three homogeneous neuron populations. To be able to illustrate key principles of disinhibitory networks, we have made several simplifying assumptions that were necessary to keep the number of free model parameters as low as possible. First, we have assumed that all cells in a population share the same properties (e.g., spiking thresholds, reset potentials, etc.). It would be interesting to test the impact of a larger cell-to-cell variability, and to include different subpopulations of *P*, *B*, and *A* cells. For example, it has recently been shown by Hunt et al. (2018) that the population of CA3 pyramidal cells can be divided into two groups of preferentially regular spiking and bursting neurons, and it has been hypothesized that the two classes play different roles in SWR initiation. This feature could be incorporated in the model once more is known about the embedding of these and other cells in the local circuit. Furthermore, we have used the standard assumption of random connectivity in the spiking network, which does not take into account distance-dependent connection probabilities and connectivity motifs that have been observed in biological networks (Song et al., 2005; Perin et al., 2011; Rieubland et al., 2014; Guzman et al., 2016; English et al., 2017). Such structured connectivities could be used to explain a number of experimental features. Bazelot et al. (2016) showed that SWR events can be triggered by driving a single pyramidal cell to spike (even a single action potential can be sufficient). This result cannot be replicated in the model, where the activation of at least ∼20-30 pyramidal cells is needed to elicit a SWR event. This limitation of the current model is because of the large size of the homogeneous pyramidal cell population, in which each neuron contributes little to the depolarization of connected cells. However, if cells were connected in a nonrandom fashion, it could be possible for a pyramidal cell with a large number of postsynaptic targets to be the initiator of a SWR. Inhomogeneous networks could also replicate the experimental finding that only up to 50% (Ylinen et al., 1995; Ellender et al., 2010) or ≈ 17% (Hájos et al., 2013) of pyramidal cells are involved in a single SWR event. In our homogeneous network, virtually all pyramidal cells participate in every SWR event. Depending on the differential embedding of cells in the network, fewer cells could be recruited in each event. This aspect of cell participation is linked to sequence replay during SWRs *in vivo*. If cells were organized in small-size clusters (cell assemblies) of strongly connected cells coding for a specific memory, only the assemblies related to the currently reactivated memory would be active in a given SWR event, thus lowering the proportion of recruited cells during a single event. This approach has been investigated by Chenkov et al. (2017) in CA3 networks comprising one excitatory and one inhibitory population, but a replication of this approach using inhomogeneous three-population disinhibitory networks is far beyond the scope of the work presented here.

Overall, our model contributes to a fundamental understanding of the role of interneurons in SWR generation. Although in this study we focused on the CA3 region to create a biologically realistic network, our model can be used to test the mechanisms underlying SWR generation in other areas (as CA1, CA2, subiculum, etc.). We predict that the disinhibitory motif is a general principle that governs the organization of hippocampal microcircuits.
